# Extraction of persistent lagrangian coherent structures for the pollutant transport prediction in the Bay of Bengal

**DOI:** 10.1038/s41598-024-58783-4

**Published:** 2024-04-16

**Authors:** V. Trinadha Rao, V. Suneel, Venkata Sai Gulakaram, Chilukuri Lakshmi Sravani

**Affiliations:** 1 CSIR-National Institute of Oceanography, Dona Paula, 403004 Goa India; 2 Academy of Scientific and Innovative Research (AcSIR), Ghaziabad, 201002 India; 3 Ministry of Earth Sciences, ESSO-Indian National Centre for Ocean Information Services (INCOIS), Hyderabad, 500090 India

**Keywords:** LCS, HYCOM, Eddies, Accumulation, Pollutants, Trajectory, Prediction, Bay of Bengal, Environmental sciences, Ocean sciences

## Abstract

Lagrangian Coherent Structures (LCS) are the hidden fluid flow skeletons that provide meaningful information about the Lagrangian circulation. In this study, we computed the monthly climatological LCSs (cLCS) maps utilizing 24 years (1994–2017) of HYbrid Coordinate Ocean Model (HYCOM) currents and ECMWF re-analysis winds in the Bay of Bengal (BoB). The seasonal reversal of winds and associated reversal of currents makes the BoB dynamic. Therefore, we primarily aim to reveal the cLCSs associated with seasonal monsoon currents and mesoscale (eddies) processes over BoB. The simulated cLCS were augmented with the complex empirical orthogonal functions to confirm the dominant lagrangian transport pattern features better. The constructed cLCS patterns show a seasonal accumulation zone and the transport pattern of freshwater plumes along the coastal region of the BoB. We further validated with the satellite imagery of real-time oil spill dispersion and modelled oil spill trajectories that match well with the LCS patterns. In addition, the application of cLCSs to study the transport of hypothetical oil spills occurring at one of the active oil exploration sites (Krishna-Godavari basin) was described. Thus, demonstrated the accumulation zones in the BoB and confirmed that the persistent monthly cLCS maps are reasonably performing well for the trajectory prediction of pollutants such as oil spills. These maps will help to initiate mitigation measures in case of any occurrence of oil spills in the future.

## Introduction

Surface Ocean currents play a vital role in simulating the trajectories of oil spills in the seas; therefore, the better trajectory prediction requires realistic currents. Nonetheless, the spatio-temporal gridded in situ measurements are sparse and only available for a particular location. Therefore, for extensive basin-scale studies, one must rely on a simulated model or satellite-derived currents. The uncertainties and errors in those simulated currents may alter the trajectory prediction of any pollutants. In this study, an attempt has been made to yield the improvised Lagrangian transport patterns using a Lagrangian Coherent Structures (LCS) method. Major oil spill accidents, oil rig blow-ups, volcanic eruptions, tsunamis, etc., are some of the global epidemics causing the release of various pollutants into the environment (land, ocean, and atmosphere). Whenever such incidents occur, immediate action is required to track the transport of released pollutants. The transportation of oil products has increased immensely worldwide due to the increased industrialization, mainly through marine waters^1^. It is estimated that about two million tonnes of oil annually enter the marine environment^2^. For instance, a major oil spill occurred off the Mumbai coast, the west coast of India, on August 07, 2010 (collision between the ships MSC Chitra and MV Khalijia), causing a spill of 800 tonnes of oil. In the same year, 2010, the explosion at the Deepwater Horizon (popularly known as the British Petroleum oil spill disaster) rig in the Gulf of Mexico caused massive effusive of oil on the seafloor, marked as the largest oil spill in US history. After that, on January 28, 2017, two ships, BW Maple and MT Dawn Kanchipuram, collided near the Ennore Port, Chennai, on the southern coast of India, in the Bay of Bengal (BoB), releasing approximately 200 tonnes of heavy fuel oil (INCOIS, 2017). On August 25, 2021, another major oil spill occurred near the Mauritius coast and led to 1000 tonnes into Indian Ocean waters^3–5^.

The released hydrocarbons from the spills could also enter the food chain and magnify their effect, causing health hazards to marine biota and humans ^6^. Therefore, it is essential to understand the trajectory of oil spills and the prediction of their movement for effective combating and monitoring. This will undoubtedly help to prevent the menace of oil pollution in the coastal regions and marine species.

Several oil spill models are used worldwide to simulate the trajectories of oil spills caused by various sources. Desa et al.^7^ have predicted the hypothetical spill trajectories over the Gulf of Kachchh (Northeastern Arabian Sea), but they could not validate the model results due to the non-availability of actual spill data. Vethamony et al.^8^ have used the MIKE21 model to simulate the trajectories of actual oil spill incidents that occurred off the Goa coast, Arabian Sea. Though this study's simulations match well with the in situ observation, this model has limitations as it works better only in coastal regions. The GNOME (General NOAA (National Oceanic Atmospheric and Administration) Operational Modelling Environment) oil spill model has also been used to simulate the oil spill trajectories in the Arabian Sea and Bay of Bengal basins^9^. All these models require various input data (oil spill scenario details, oil properties, met-ocean data, and output requirements) for the model, and the simulated trajectories need to be validated further with the in situ observations. In addition, the ambiguity in the forecasting of oil trajectory comes due to discrepancies in the input fields (initial conditions, met-ocean data) and sparse observational data^10^. Further, identifying the source and transport of pollutants in near-shore regions is challenging due to the complex flow fields in the coastal waters.

Despite the availability of various data products in recent times, the oil spill forecasts are still uncertain since the underlying ocean flow and the associated transport are chaotic. Therefore, it is high time for an advanced alternate mechanism to quickly simulate an accurate oil spill transport pattern. Lagrangian Coherent Structure (LCS) method is a powerful tool for analyzing fluid flows and predicting the motion of passive particles, such as oil spills. LCSs are the locally most robust repelling or attracting material lines representing the cores of Lagrangian patterns that an ideal tracer cannot cross. It is effective over existing oil spill models in several ways. (i) LCS analysis can identify the most critical structures in a flow field that control the transport of particles and, therefore, can provide more accurate information about the transport/drift of an oil spill. (ii) It is relatively simple to implement and does not require as many assumptions as traditional oil spill models, such as steady-state flow, homogeneous fluid, particle size, density, etc. Garcia-Garrido et al.^11^, identified the date and arrival point of the oil to the coast after a fishing ship accident occurred. Gouveia et al.^12^ have identified the most likely origin of unknown oil/tar beaching along the 4000 km of Brazil's coast using the cLCS. They have also highlighted that cLCS have depicted two different transport patterns illustrated by the synthetic drifters and iSphere trajectories.

Several diagnostic tools were proposed to interpret the formation of the Lagrangian pattern in unsteady flows^13–16^. Wherein the finite-time Lyapunov exponents (FTLE) have recently received much attention. The ridges of the FTLE field are routinely considered indicators of hyperbolic (i.e., attracting or repelling) Lagrangian Coherent Structures (LCSs)^17,18^. Simulation of climatological Lagrangian Coherent Structures (cLCS) is effective in emergency response and preparation of contingency plans for oil spills^19–21^ performing rescue operations, assessment of larval and fish connectivity, predicting the dispersion and destination of marine debris and other waste pollutants^12^. The LCS was also used to assess the Deepwater Horizon oil spill event in the Gulf of Mexico (e.g.^22–24^ which found that the attractive LCS closely matches the SAR observed oil spill in the Mediterranean Sea. Overall, the LCS method provides a more accurate, flexible, and straightforward approach to predicting the behaviour of oil spills in different environments. Thus, the identification and quantitative description of climatological LCS is expected to improve the effectiveness of the future emergency response to oil spills and contingency planning^12^. The method of computing cLCS was recently introduced by Duran et al.^25^, who extracted the Lagrangian transport patterns from large velocity time series (12 years of data).

To find dominant patterns of behavior from scalar and vector datasets, empirical orthogonal function (EOF) analysis has been widely employed in meteorology and oceanography^26–29^. Several studies of EOFs have been applied to a variety of climate science (Meteorology and Oceanography) research, including those on winds^30,31^, currents^32–35^, and sea surface temperature ^36^. EOFs are used to break down data fields produced by spatiotemporal dynamical systems into a small number of constituent leading EOF modes, which allow for the reconstruction and description of the dynamical system's complete data fields. To put it briefly, it is possible to reduce a large-dimensional dynamical system to fewer dimensions without sacrificing its essential information. It is feasible to switch an EOF analysis into a CEOF (Complex Empirical Orthogonal Function) analysis when two scalar fields are connected to a variable, such as u and v components of surface currents. Therefore, in this current study, we have employed CEOF analysis of ocean surface currents data to understand the significance of dominant features and their relation with cLCS in BoB.

The North Indian Ocean is dynamic due to the seasonally reversing winds that modulate the seasonal reversing currents in the Arabian Sea and the Bay of Bengal. The eastward-flowing Summer Monsoon Currents (SMC) from May to September and westward-flowing Winter Monsoon Currents (WMC) from November to February are prominent in these basins^37^. The East India Coastal Current (EICC), also known as the western boundary current of the Bay of Bengal^38–42^ flows along the east coast of India. The impact of the transient monsoon system alters the EICC direction twice a year^43,44^, flowing northward during February–May and southward during October–December. During the Indian Summer Monsoon period (June–September), the boundary current is not consistent as the western BoB is also dominated by high eddy activity significantly at northern and southwestern BoB and offshore Visakhapatnam, which are responsible for the discontinuity observed in EICC during Indian Summer Monsoon period^45^. The accurate depiction of surface flows in current systems dominated by instabilities and intense mesoscale activity is particularly challenging, and therefore, the goals of this study are (i) To extract and depict the monthly Lagrangian transport patterns over the highly dynamical basin of the BoB from a long (24 year) HYCOM surface currents data using the LCS^25,46^; (ii) To analyze the Lagrangian transport patterns associated with the dynamic Western Boundary Current (WBC, also known as the EICC) and its associated eddies in the western BoB; (iii) Comparison of the computed cLCS with the dispersion of actual oil spill incident that occurred about two nautical miles (13.2282 ^∘^ N 80.3633 ^∘^ E) off Kamarajar Port, Ennore on January 28, 2017^9^; (iv) To depict the transport pattern of hypothetical oil spills occurring near Krishna-Godavari basin fields in the BoB based on the derived cLCS patterns.

### Data and methodology

#### Surface current, wind and drifter velocities

The long-term surface current velocities are the only input data for computing the cLCS. Earlier studies by Gough et al.^47^ and Maslo et al.^52^ have used the velocities simulated with NEMO (Nucleus for European Modelling of the Ocean) and ROMS (Regional Ocean Modeling System), respectively, while Duran et al.^25^ used an operational HYCOM (Hybrid Coordinate Ocean Model) simulations to compute the cLCSs. Nevertheless, all these studies accurately depicted the predominant transport patterns through cLCS. In this study, we used 24 years (1994–2017) of high-resolution global HYCOM (Hybrid Coordinate Ocean Model)^53^ ocean currents for computing the cLCS in the Bay of Bengal. HYCOM is one of the most efficient models to simulate reliable oceanic currents worldwide for operational oceanography^54–56^. HYCOM simulation currents were used in several studies, mainly to simulate the oil spill trajectories in the Indian Ocean as well as other oceans^57–59^. HYCOM is a primitive equation Ocean General Circulation Model, which includes wind and surface heatflux. Therefore the velocities simulated by this model include both geostrophic and ageostrophic (Ekman flow) contributions, such as wind-driven circulation and mixing^53,60^. Therefore, this study uses the freely available long-term currents derived from the HYCOM model with a spatial resolution of 0.08° and a temporal resolution of 3 h (later converted into daily velocity data) from the HYCOM model dataset. Similarly, the surface re-analysis winds obtained for 24 years (1994–2017) from the European Center for Medium-Range Weather Forecasts (ECMWF) has a temporal resolution of 6 h and a spatial resolution of 1/8° in latitude and longitude. The acquired 24 years (1994–2017) of HYCOM currents and winds are used to create the daily climatological surface current velocities and winds.

The satellite-tracked surface drifting buoys data for the period of one month, January 2015 is obtained from the NOAA Global Drifter Programme. The Atlantic Oceanographic and Meteorological Laboratory (AOML) has applied quality control procedures and interpolated them to hourly intervals^61^.

#### Lagrangian coherent structures

The LCSs were extracted from the daily climatological surface current velocities, and it is the first attempt to construct the monthly LCS maps over the entire BoB basin. The methodology of the computing cLCS is adopted from Duran et al.^25,62^, and the same is followed by Gough et al.^47^ and Gouveia et al.^12^ and also briefly described here. LCS are structures in fluid flow that reveal the most persistent patterns of fluid motion, giving a clear and new way of understanding the complex Lagrangian transport. The LCS shows the collective information of the primary mean flow. The establishment of LCS starts with a velocity equation for fluid particles.

1 $$\tfrac{{{\text{dx}}}}{{{\text{dt}}}}{\text{ = V}}\left( {\text{X, t}} \right)$$

Where **V**(x, t) is the two-dimensional velocity of the fluid, by solving this equation with initial position x_0,_ at initial time t_0_, the particle will reach position x at time t_1._ Thus, the flow map of the particles from their initial position to their final location is defined as follows.

2 $$F_{{(t_{0} )}}^{{(t_{1} )}} (x_{0} ) = x(t_{1} ;x_{0} ,t_{0} )$$

Once the flow map of the particles has been obtained, the next step is computing the Cauchy-Green deformation tensors. The Cauchy-Green tensors play an essential role in the identification of LCS. The eigenvalues and eigenvectors of the Cauchy-Green tensors provide information about the stretching and compression of fluid particles in the flow. The regions of the flow where the eigenvectors of the Cauchy-Green tensors align with the flow direction are known as repelling LCS, while the regions where the eigenvectors align perpendicular to the flow direction are known as attracting LCS. The Cauchy-green tensors are measured based on the following matrix.

3 $$C_{{t_{0} }}^{{t_{1} }} \left( {x_{0} } \right) = \left[ {\nabla F_{{t_{0} }}^{{t_{1} }} \left( {x_{0} } \right)} \right]^{T} \nabla F_{{t_{0} }}^{{t_{1} }} \left( {x_{0} } \right)$$

where, $\nabla {F}_{{t}_{0}}^{{t}_{1}}({x}_{0})$ denotes the Jacobian matrix solution of velocity Eq. (1).

The eigenvalues λ_t_ and corresponding unit eigenvectors ξ_t_ of the tensor ${C}_{{t}_{0}}^{{t}_{1}}\left({x}_{0}\right)$ are defined by a relation.

4 $$C_{{(t_{0} )}}^{{(t_{1} )}} (x_{0} )\xi i(x0) = \,\lambda i(x0)\xi i(x0)|\xi i(x0)| = 1,\,\,i = 1,2$$

Here λ and ξ represent the Eigen values and Eigen vectors. As an outcome, the Eigen values and Eigen vectors satisfy 0 < λ_1_(x_0_) ≤ λ_2_ (x_0_) and ξ_1_(x_0_) ⟂ ξ_2_(x_0_)_,_ respectively.

A hyperbolic LCS at time t_0_ that attracts the neighboring fluid from t_0_-T through t_0_ (T > 0) is a prepared material curve x_0_ (s) such that.

5 $$\tfrac{{d_{{x_{o} }} }}{ds} = \, \xi_{1} (X_{0} )$$

Here ξ_1_ (x_0_) is tangent to x_0_ (s) and along which

6 $${\uprho }_{{(t_{0} )}}^{{(t_{1} )}} ({\text{X}}) = \sqrt {\lambda_{2} } (X) > 1$$

Note that when the material curve satisfies (5) and (6), it normally attracts the nearby fluid from t_0_–T to t_0_, which indicates that the repels fluid in the computation direction, t_0_ to t_0_-T, which are referred to as squeezlines^21^. It means a curve s → X(s) satisfies Eqs. (5),(6). The most attractive LCSs in forward time are those with the largest back-in-time normal repulsion ${\uprho }_{{t}_{0}}^{{t}_{1}}$(X). All integrations were carried out using the fourth-order Runge–Kutta method. The computational domain covers the Bay of Bengal region by the mean grid spacing of 2.1 km. To evaluate the centred derivatives, an auxiliary grid is employed, featuring 4 points separated by 0.1 km and centered at each grid point of the main grid is used to evaluate the centered derivatives.

#### Climatological lagrangian coherent structures (cLCS)

The methodology for the computation of cLCS is taken from Duran et al.^25^. In brief, the methodology consists of two steps. The first is calculating the daily velocity climatology. Thus, the flow climatology for each day is the average of 24 years (1994–2017) of HYCOM currents data. With this flow, we computed the backward-in-time Cauchy-Green (CG) tensor fields on a sliding 7-day time window (T = − 7). The justification for a 7-day time window is that a few days to a week is a critical time scale for oil spill response which is the primary interest of this study. The next step consists of averaging several CG tensors. We computed CG tensors considering the fixed time scale T = − 7 days, with initial times t0 ∈ {8, 10, 12, …,30} days with an increment of 2 days. The monthly mean CG tensor is the average CG tensor. The averaging of CG tensors destroys the invariance property seen in the LCS computed from the daily climatological surface currents^47^. These time-averaged CG tensors are used to compute LCSs. These LCS follow as squeeze lines of the monthly averaged Cauchy-Green tensor fields referred to as cLCS. This method delivers comprehensive and temporally smoothed flow patterns for every month^25^. The procedure described in “Surface current, wind and drifter velocities” and “Lagrangian coherent structures” is repeated with wind data to derive the wind induced cLCS.

An attempt is made to compare the LCS randomly chosen for January 2015 with the surface drifters trajectories (Fig. S1, Supporting Information). Four drifters (nos #116349, #35777, #138409, and #138410) are available for this month in the BoB region. The figure depicts that the drifters #116349 and #35777 have followed their path more closely with the LCS. Whereas the path of drifters #138410, and #138409 has not been followed as other drifters. Though part of its path matches the LCS, a part of its trajectory has not followed the LCS. The plausible reason could be the influence of sub-mesoscale processes. The path of the drifter trajectory can be influenced by the local mesoscale processes. The western BoB is known as one of the eddy-dominated regions ranging from sub-mesoscale to mesoscale. This may be the reason for the drifter #138410 trajectory has a loop around 15° N. Since the sub-mesoscale processes are not well included in the HYCOM currents from which the LCS are generated, this mismatch may be observed. On the whole, the LCS is reasonably matched with a drifter trajectory other than the eddy-dominant region.

#### Complex empirical orthogonal function (CEOF) analysis

CEOFs are used to analyse the spatial patterns and temporal variability of ocean currents^48^. While CEOF modes represent spatial patterns which capture the coherent structures and variability of ocean currents, the corresponding principal component time series represents the temporal evolution of these spatial patterns over time^49^.

The PCA (Principal component Analysis) with CEOFs was examined by^50^ and^51^ with reference to the wind vector field derived from the zonal and meridional components u and v of the wind field. Like the wind field, the meridional current component v and the zonal current component u are the two scalar fields associated with the Hycom surface ocean currents.

The HYCOM daily currents data of BoB from 1994 to 2017 is resampled into monthly data. Then, CEOF analysis is applied to each monthly dataset separately. Ocean current data is represented as complex values.

$${\text{z}}={\text{u}}+{\text{iv}}$$

Here, z represents the complex-values data at a particular spatial point and time point, while i represent $\sqrt{-1}$. u and v represent zonal and meridional components of ocean currents, respectively. Currents data is organised into a complex matrix Z, which is represented as.

$$Z\, = \left[ {\begin{array}{*{20}c} {z_{11} } & {\begin{array}{*{20}c} {z_{12} } & \ldots \\ \end{array} } & {z_{1n} } \\ {\begin{array}{*{20}c} {z_{21} } \\ \vdots \\ \end{array} } & {\begin{array}{*{20}c} {\begin{array}{*{20}c} {z_{22} } & \ldots \\ \end{array} } \\ \vdots \\ \end{array} } & {\begin{array}{*{20}c} {z_{21} } \\ \vdots \\ \end{array} } \\ {z_{m1} } & {\begin{array}{*{20}c} {z_{m2} } & \ldots \\ \end{array} } & {z_{mn} } \\ \end{array} } \right]$$

Where m is the number of spatial points, and n is the number of time points. Covariance matrix C is computed for matrix Z and eigenvalues (${\uplambda }_{{\text{i}}}$) and eigenvectors (${{\text{v}}}_{{\text{i}}}$) of matrix C are calculated using

$${\text{Cv}}_{{\text{i}}} = {\uplambda }_{{\text{i}}} {\text{ v}}_{{\text{i}}}$$

The eigenvalues are sorted in descending order, and the ${{\text{i}}}^{{\text{th}}}$ CEOF mode (${{\text{CEOF}}}_{{\text{i}}}$) is given by the ${{\text{i}}}^{{\text{th}}}$ eigenvector ${{\text{v}}}_{{\text{i}}}$ corresponding to eigenvalue ${\uplambda }_{{\text{i}}}$. These modes capture the spatial patterns of the current data over the BoB.

The variance fraction indicates the proportion of total variance explained by each CEOF mode. The variance fraction (${{\text{VF}}}_{{\text{i}}}$) for the ${{\text{CEOF}}}_{{\text{i}}}$ can be calculated as

$$VF_{i} = \tfrac{{\lambda_{i} }}{{\Sigma_{i = 1}^{m} \lambda_{i} }}$$

Leading CEOF modes explain more variance in the dataset. Here, CEOF modes are expressed as the correlation between the corresponding principal component time series (PCs) and the time series of the input data at each grid point. High correlation values at certain grid points indicate that those regions contribute significantly to the spatial pattern represented by the corresponding CEOF mode.

#### Sentinel-1 satellite imagery

In this current study, we have used the Sentinel-1 SAR (Synthetic Aperture Radar) C band images for 2017 along the Krishna–Godavari (KG) basin and near the oil spill accident region. This Sentinel-1 satellite carries a C band SAR sensor with a frequency of 5.405 GHz and a wavelength of 5.5 cm. We acquired 61 GRD (Ground range detected) images, of which 24 images contained 57 oil spills along this region with 10*10 m resolution. The best suitable GRD product covers an overall 250 km spatial length. The acquired satellite images are processed in the ESA-SNAP toolbox and QGIS to identify and group the oil spills according to their location^63^.

#### GNOME oil spill model

The General NOAA Operational Modeling Environment (GNOME) is a freely available simulation model that NOAA's Hazardous Materials Response Division developed. This model has been widely used and validated based on observations of several oil spill events worldwide [65,64,9,4]. This study uses the GNOME model to simulate the oil spill incident that occurred off Ennore Port Chennai due to a collision between BW Maple and MT Dawn Kanchipuram. The model is forced with 200 metric tonnes of medium crude oil, 3% windage, and 100,000 cm^2^/sec diffusion. The model was simulated for two days, from January 28, 2017- 00:00 to January 29, 2017- 23:59, with only currents and winds, currents, and diffusion effects. The HYCOM currents that are described in “Surface current, wind and drifter velocities” are used in this study. Whereas the ECMWF ERA5 global reanalysis winds obtained for the simulation period were used. The zonal and meridional wind data acquired from ECMWF has an excellent spatial resolution (0.25 × 0.25 degrees, approximately 28 km) and temporal resolution (hourly)^66,67^.

## Results and discussion

We demonstrated the extracted LCS in the BoB and verified that the velocity climatology conserves meaningful transport patterns portrayed by cLCS. We first compared the cLCS with the SAR satellite image of an oil slick in the same region. In this study, for the first time, the climatological Lagrangian Coherent structures that are associated with the monsoonal (coastal) currents were yielded and explained with respect to different seasons such as (1) SW monsoon, (2) NE monsoon, (3) Pre and post-monsoon seasons.

### Monthly climatological maps of Lagrangian Coherent Structures in the Bay of Bengal

#### cLCS during the Indian southwest monsoon period

**Figure 1:**
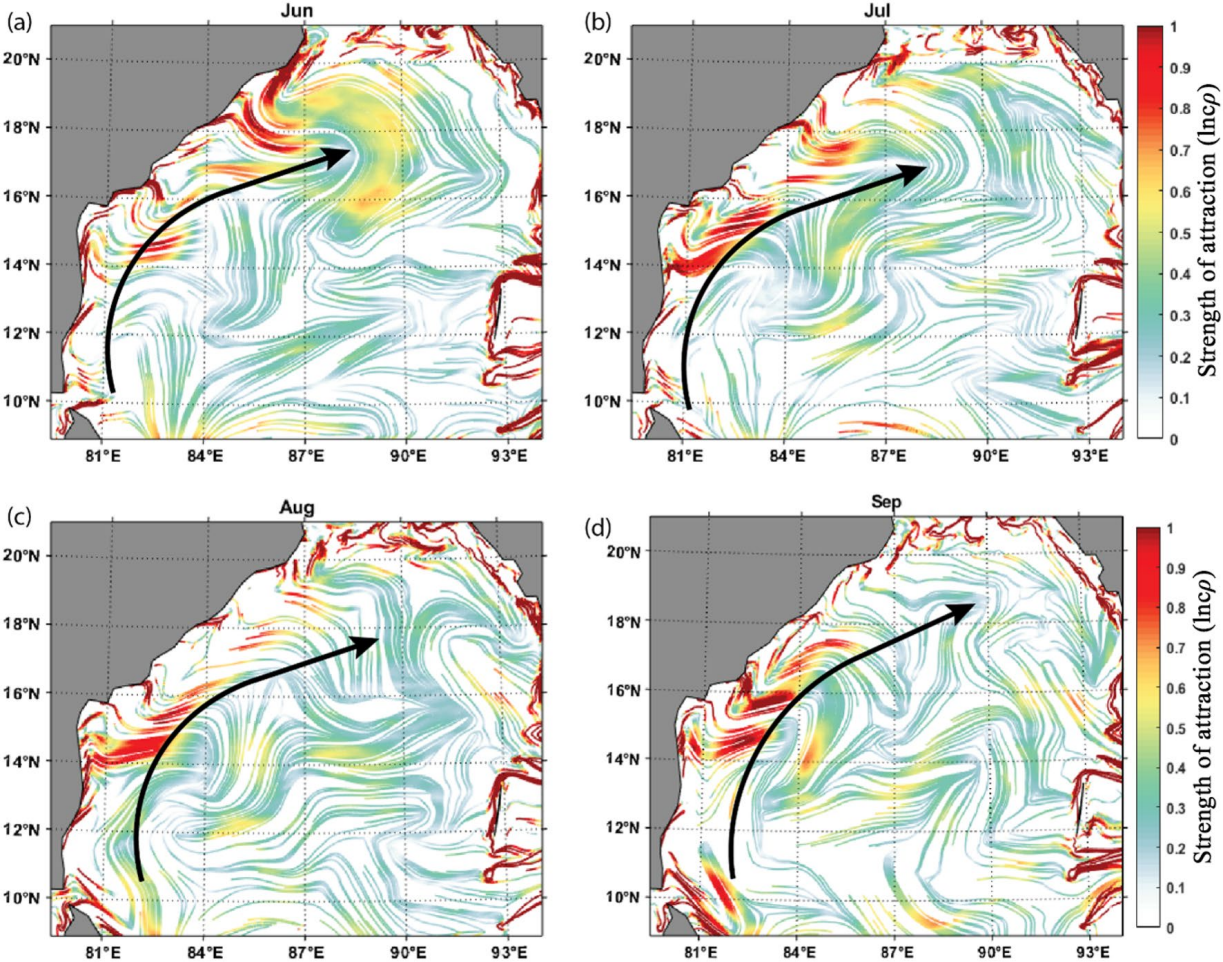
The constructed monthly cLCS maps during the Indian Summer Monsoon (SM) season over the Bay of Bengal. (**a**) June, (**b**) July, (**c**) August, (**d**) September. The colour bar in the figure represents the variation of climatological attraction strengths in lncρ, and the overlaid black arrow indicates the EICC direction in general during the SW monsoon period.

The BoB is forced by the semiannual reversal of monsoon winds from May to September (southwesterlies) and from December to February (northeasterlies). These seasonal winds are essential in determining the upper ocean circulation in the Bay of Bengal^68^. Therefore, in the summer monsoon season, the prevailing winds over BoB are towards the northeast. The EICC reverses seasonally in response to the seasonal reversing winds^37,38,68,69^. In general, the surface circulation during southwest monsoon is clockwise over BoB. An anticyclonic gyre pattern is seen during the southwest monsoon, with a northeastward flowing EICC forming the western boundary current of the basin-wide anticyclonic gyre. Figure 1 (a,b,c, and d) depicts the monthly maps of cLCSs from June to September, representing the southwest monsoon season. It is evident from this figure that the cLCSs are strong all along the coastal boundary of the basin, more significantly along the east coast of India by curving eastward. These strong cLCSs along the East Coast are primarily attributed to the EICC. The relatively weak cLCSs are in the central BoB, away from the coast. Interestingly, during June, the hook-like cLCS pattern is seen between 17 to 18° N, curving eastward towards offshore in a clockwise direction. This is the region of highly active eddy genesis^45^. Thus, these cLCS results also indicate that the strong seasonal and mesoscale processes yield strong cLCSs. The strength of attraction is a non-dimensional quantity and gives the intensity of the attraction or stretching of fluid elements in a flow over a finite time interval in logarithmic units.

#### cLCS during the Indian winter monsoon period

**Figure 2:**
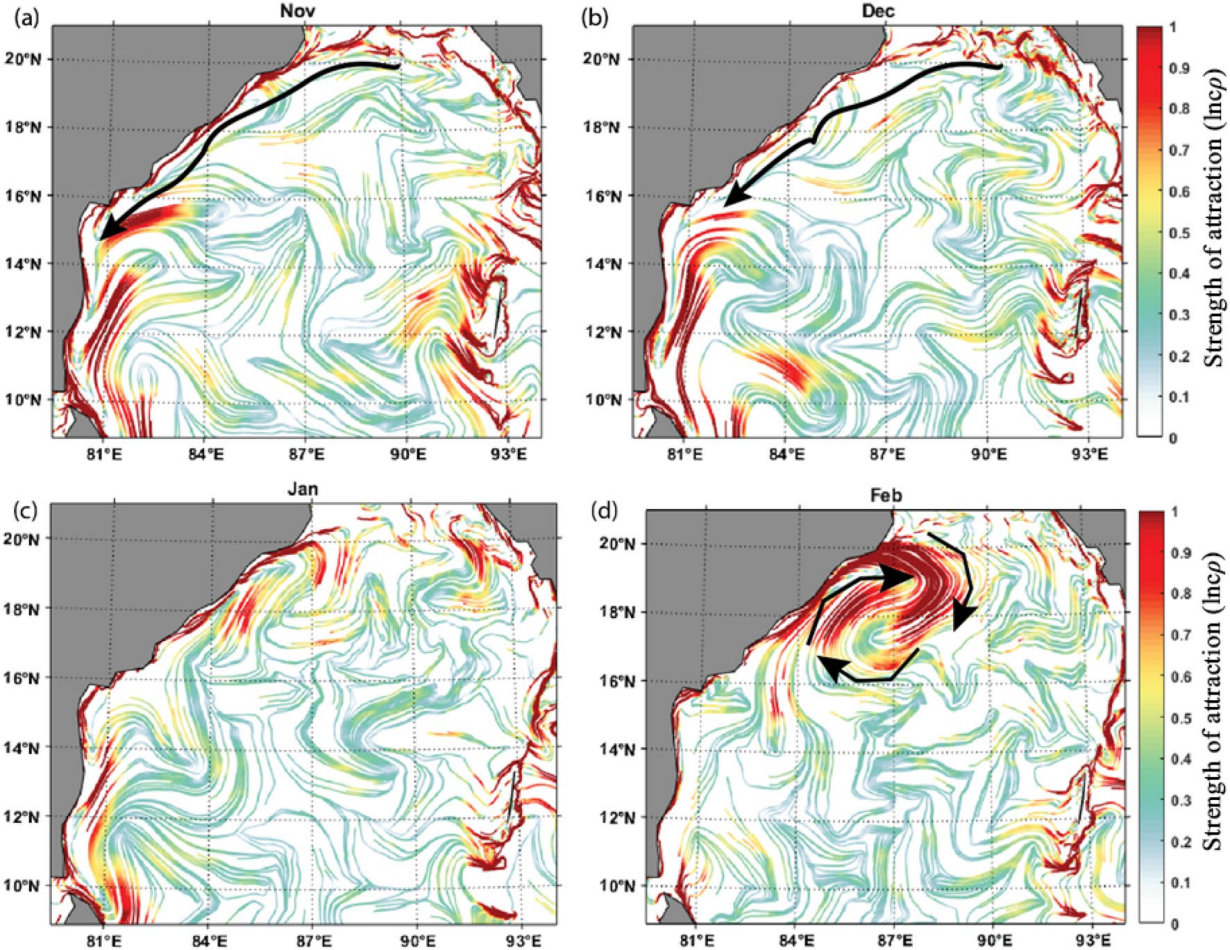
The constructed monthly cLCS maps during the Indian Winter Monsoon (WM) season over the Bay of Bengal. (**a**) November (**b**) December (**c**) January (**d**) February. (The colour bar in the figure represents the variation of climatological attraction strengths in lncρ). The overlaid black arrow in Fig (**a**),(**b**) indicates the EICC direction. The clockwise pattern of black arrows in Fig. (**d**) Indicates the anticyclonic eddy.

During the winter monsoon season, the circulation pattern over BoB is anticlockwise. The EICC flows equatorward during the winter monsoon (October to February), with peak flow during November^69^. Figure 2 depicts the monthly maps of cLCSs during the winter monsoon. As seen during the summer monsoon, the cLCSs are strong along the coastal boundary and are relatively weak in the open ocean. As depicted in Figs. 2a,b, the strong cLCSs along the east coast of India during November–December are attributed to the strong equatorward EICC induced by the local and remote forcing. The cLCSs are slightly weakening during January, and by February, they are significantly weak south of 16^o^N. Nevertheless, a strong anticyclonic cLCSs pattern is seen between 18–20° N during February. This is due to the formation of anticyclonic eddy over there in February. This is the region off the Visakhapatnam Coast, where the dominant occurrence of anticyclonic eddy activity is relatively high^45,70^. Thus, it is evident that cLCSs could capture the quasi-steady Lagrangian transport patterns associated with the mesoscale eddies and seasonal reversing western boundary current in the BoB.

**Figure 3:**
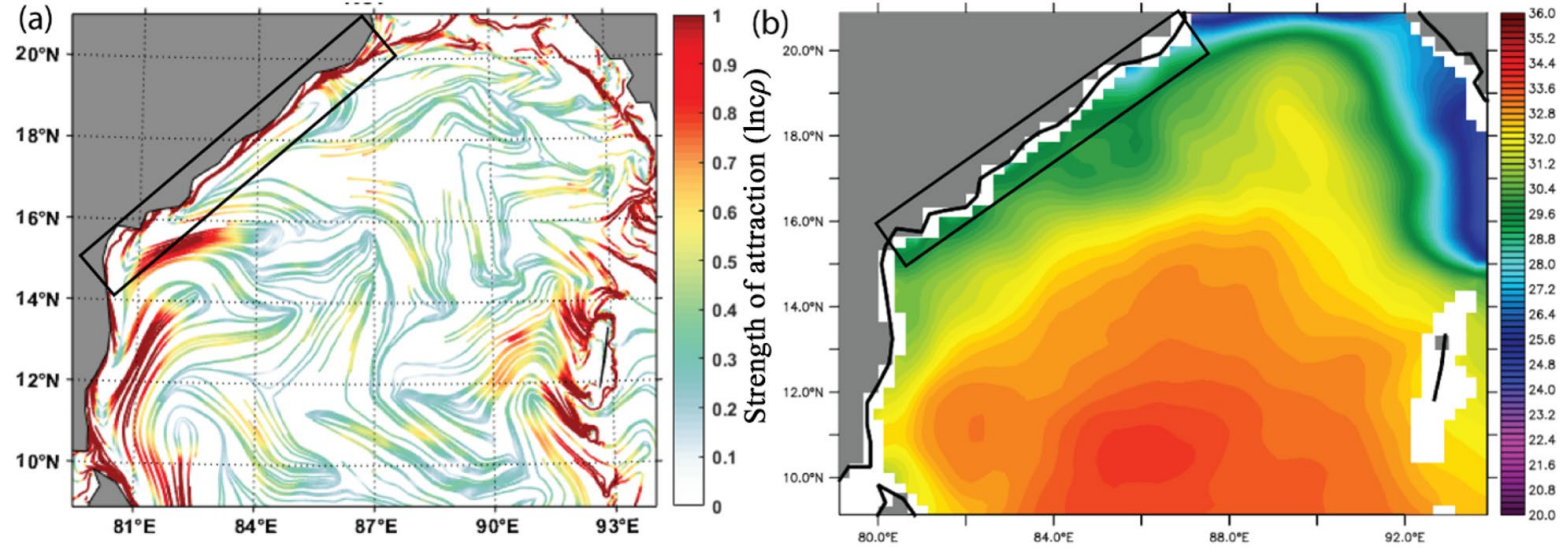
(**a**) Climatological LCSs for the November month (The colour bar in the figure represents the variation of climatological attraction strengths in lncρ) (**b**) November Climatological SSS (Sea Surface Salinity) over the Bay of Bengal for the period of 2015-present. Block rectangular box indicates the southward advection of freshwater plume primarily driven by the EICC. The corresponding strong cLCSs are also evident in this region, marked in a black rectangular box (Fig. (**a**)).

The northern BoB receives a large seasonal freshwater influx from the perennial rivers, and this basin is characterized by excess precipitation over evaporation ^71^. The accumulated fresh water is carried by the equatorward EICC soon after the southwest monsoon season^72,73^. Suneel et al., ^72^ have depicted that during the month of November, when the EICC becomes the strongest, the 'river in the sea' (transport of northern BoB freshwater plume) develops all along the east coast of India (Fig. 3b). Figure. 3a depicts the prevailing strong cLCSs all along the east coast of India during November. Thus, it further indicates that the cLCSs can also be used to understand the seasonal transport patterns of freshwater plumes in the coastal regions of BoB^74,75^.

#### cLCS during the Pre and post-monsoon periods

**Figure 4:**
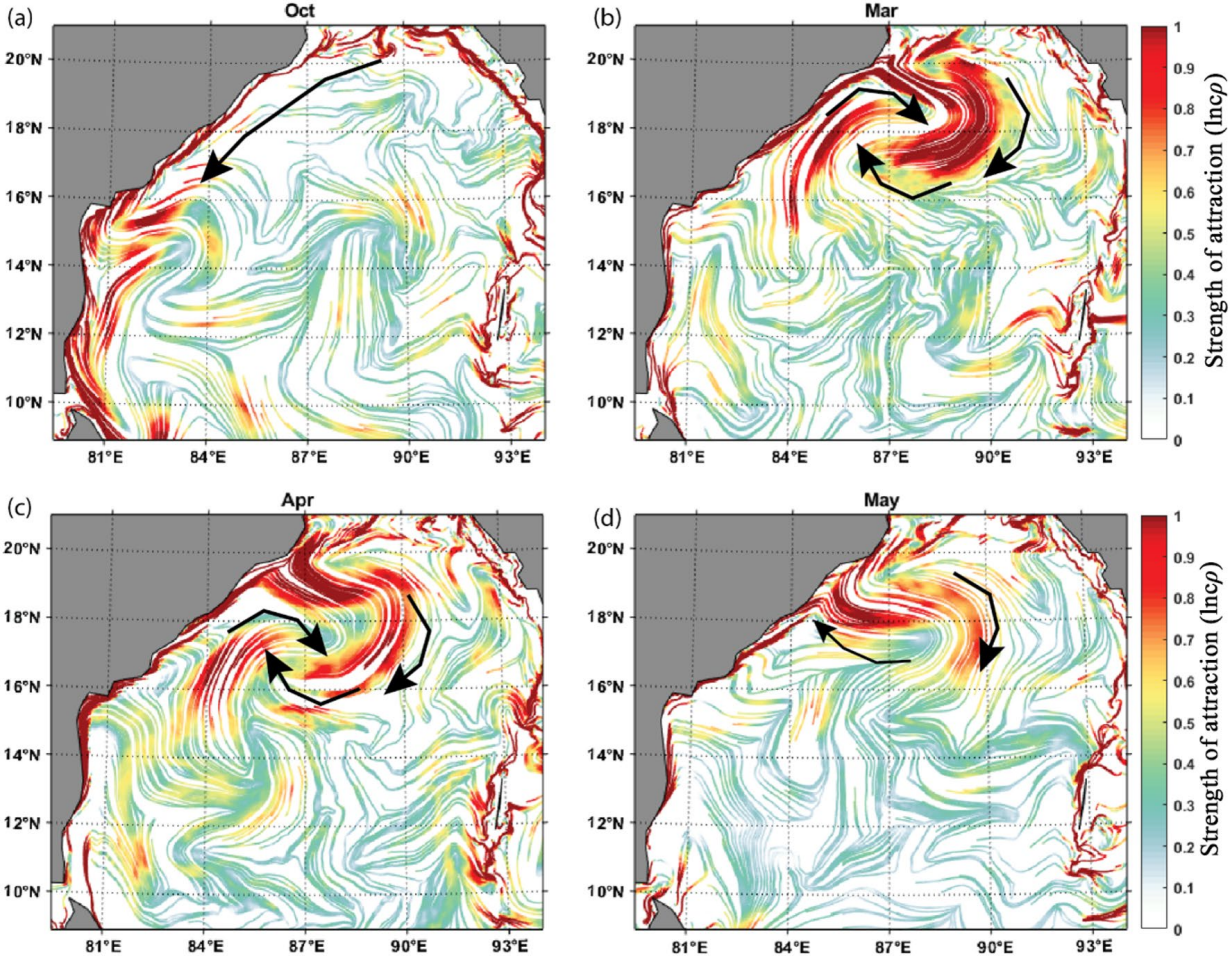
The constructed cLCSs maps during Pre and post-monsoon seasons, (**a**) October (post-monsoon), (**b**) March, (**c**) April, (**d**) May (Pre-monsoon). (The colour bar in the figure represents the variation of climatological attraction strengths in lncρ). The black arrow indicates the strong, attractive cLCSs corresponding to the anticyclonic eddy.

During pre-monsoon (March, April and May), the poleward EICC is very prominent as the western boundary of the seasonal subtropical gyre^38^. The basin-wide surface circulation forms as an anticyclonic gyre during pre-monsoon^38,76^. Recently, Mukhopadhyay et al.^77^ and Mukherjee et al.^69^, based on the in situ ADCP data, confirmed that the EICC flows poleward from February to May. Figure 4b,d show the constructed cLCSs during March, April and May, respectively. As observed in other months, the solid attractive cLCS are observed along the coastal boundary, particularly along the western BoB, attributing to the poleward EICC. The cLCSs in the open ocean are relatively weak. The equatorward EICC develops during October; in line with this current, robust cLCS patterns are also seen along India's east coast (Fig. 4a).

**Figure 5:**
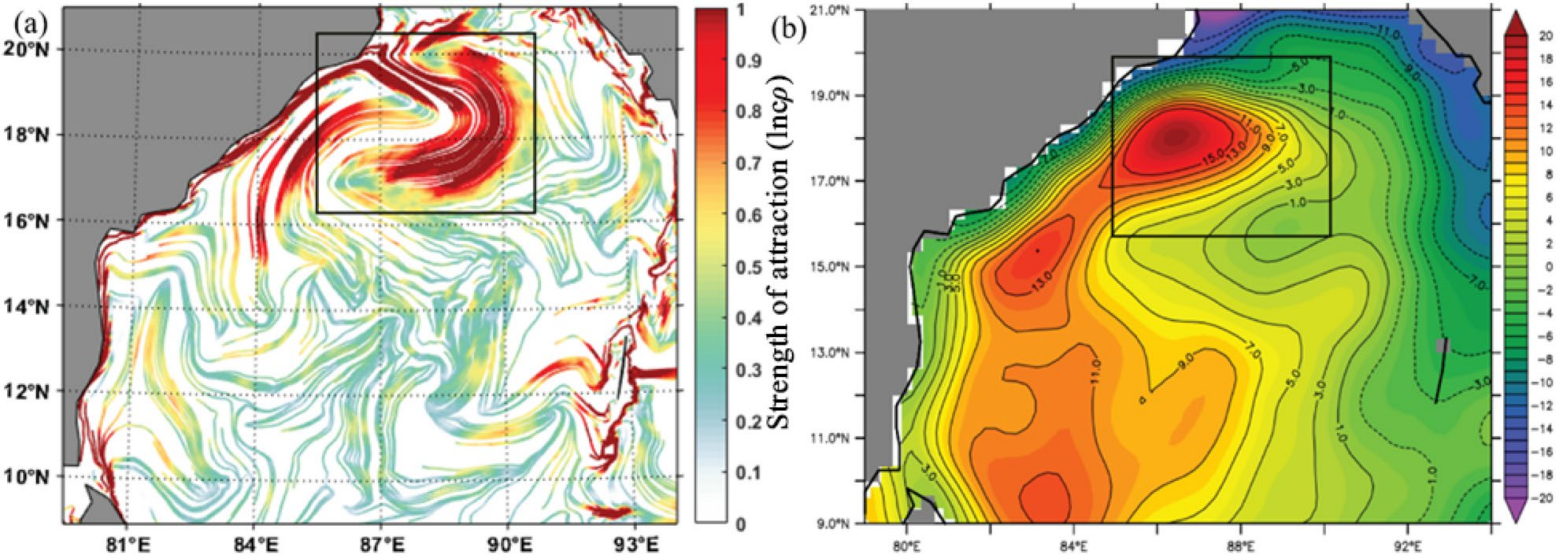
March climatological LCSs (The colour bar in the figure represents the variation of climatological attraction strengths in lncρ) (**b**) March climatological Sea level anomaly over BoB for 1994–2017. The square box inside Fig. 5a represents the strong cLCS induced by an anticyclonic eddy near Visakhapatnam, and Fig. 5b represents the presence of an anticyclonic eddy.

It was evident that during the winter monsoon period, in particular during February, the formation of an anticyclonic eddy off Visakhapatnam (18^o^–20° N) (Fig. 2d). In continuation, based on the strong cLCS pattern during March, April and May, it is evident that the strong anticlockwise cLCS pattern is seen off Visakhapatnam (18^o^–20° N) during March (Fig. 5), and it is slightly weakend during April and almost dissipated in May. Thus, it is apparent that from February to May, the genesis, growth and dissipation of an anticyclonic eddy off Visakhapatnam triggered the strong, attractive cLCS pattern over those months. Hence, this region is considered one of the accumulation zones, wherein the anticyclonic eddy could trap all the floating pollutant debris (such as oil or microplastic particles) that the EICC is transporting. Since this eddy is seen only for four months, this zone is considered the seasonal accumulation zone for floating pollutants. Nevertheless, the in situ data is needed to confirm this.

In this study, we further attempted to understand the contribution of winds to the cLCSs in the BoB. As described in Sec 2.1 and 2.2, the cLCS were also computed with the winds alone. Figure S2, in supporting information, illustrates the computed wind-induced cLCSs for all 12 months. It is evident from the figure that the strength of cLCS is almost zero in all months except October, November and December. During October, the cLCS are seen only in the northern Bay of Bengal (only the small region). Whereas in November and December, the cLCS are relatively strong and significant. This indicates that the wind contribution during the northeast monsoon, particularly during November and December, is evident. In general, the surface winds are more systematic and organized in direction than the current system in the sea; therefore, the wind-induced cLCS are more straightforward and are only significant in the eastern and northern BoB. Thus, these wind-induced cLCSs may play a role in drifting the surface transport from the eastern and northeastern BoB towards the western BoB and hence India's east coast.

#### cLCS and CEOFs (Complex empirical orthogonal functions)

**Figure 6:**
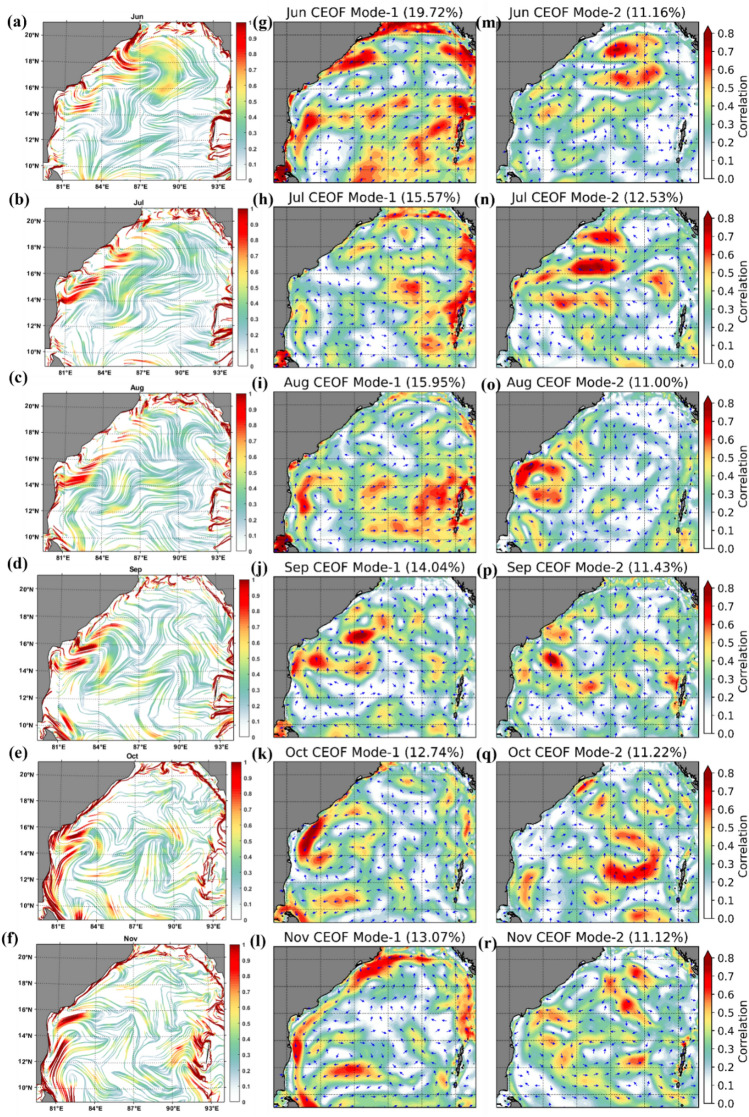
cLCS (**a**–**f**)(The colour bar in the figure represents the variation of climatological attraction strengths in lncρ) and complex EOF analysis mode-1 (g-l) and 2 (m-r) over the Bay of Bengal during 1994–2017 for the months of June, July, August, September, October and November.

The complex empirical orthogonal function analysis has been applied to surface currents in the Bay of Bengal (79–94° E, 09–21° N) from 1994 to 2017, and the two leading modes (CEOF1 and CEOF2) are illustrated (Fig. 6). The present study aims to determine the association between CEOFs and cLCS in the Bay of Bengal surface currents. Each Complex Empirical Orthogonal Function (CEOF) mode variance fraction indicates the percentage of the total variance that a particular mode accounts for.

During the SWM season, the CEOF mode-1 showed the dominant feature of currents in the coastal BoB region (Fig. 6), especially in the months of June (19.72%), July (12.53%) August (15.95%) and September (14.04%) (Fig. 6 g, i and j) than mode-2. Meanwhile, during October and November, the percentage difference between mode-1 and 2 is not very high, even though mode-1 is slightly dominant (Fig. 6 k,l,q,r). However, it is worth noting that during the months of July and August, the EOF modes-1 and 2 show the dominant transport patterns in eastern, western, southeastern and western Bay with greater than 0.4 correlation (yellow with red patches in Fig. 6 h, n, i and o) which are not very prominently seen in cLCS.

**Figure 7:**
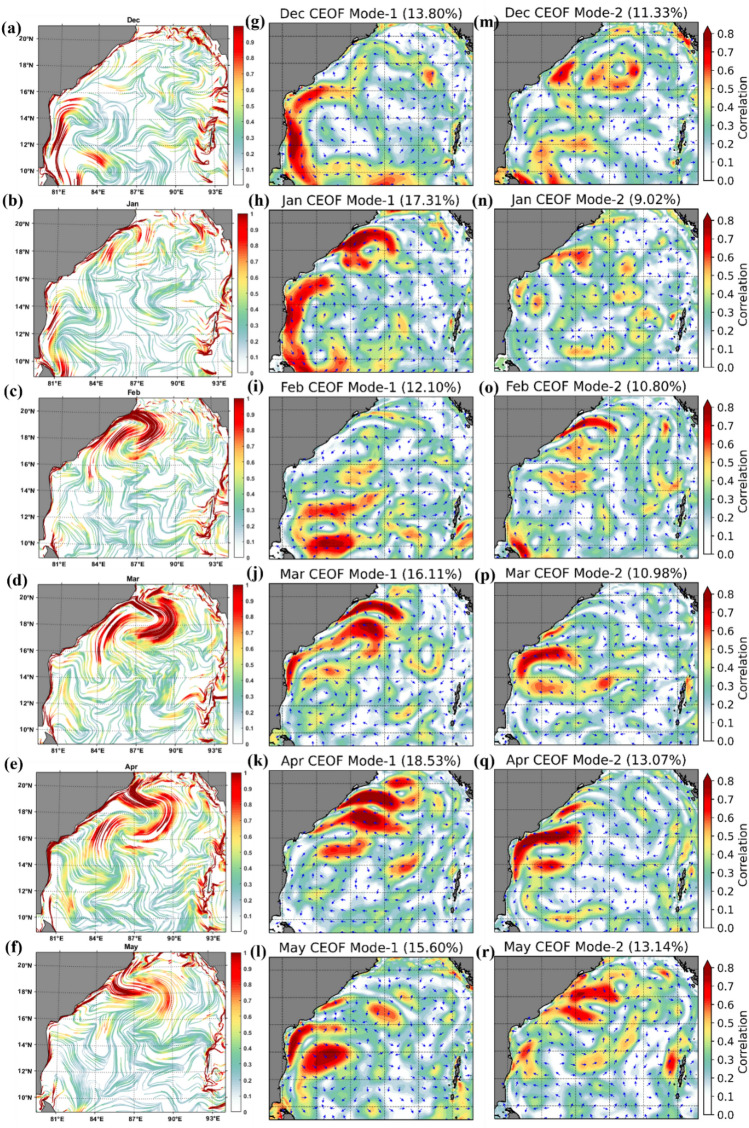
cLCS (**a**–**f**) (The colour bar in the figure represents the variation of climatological attraction strengths in lncρ) and complex EOF analysis mode 1 (g–l) and 2 (m–r) over the Bay of Bengal during 1994–2017 for December, January, February, March, April and May.

During the NEM season, the CEOF mode-1 (Fig. 6k, l and Fig. 7g, h) captured dominant features similar to those depicted by the cLCS (Fig. 7a,b) than mode-2 (Fig. 7m,n). It is significant that the cLCS are stronger along the coastal region of India during the NEM season. The EOF mode-1 also captured the dominant feature, indicating that the result of CEOF mode-1 and cLCS both captured the dominant features, indicating that the result of CEOF mode-1 and cLCS reasonably captured the dominant features during NEM. Meanwhile, the eddy features that are significantly seen in cLCS during pre-monsoon (February, March, April and May Fig. 7c,d e and f) are not very significant in EOFs (Fig. 7i,j,k,o,l,p and q). During these months, the cLCSs exhibit prominent eddy features along the western BoB (Fig. 7c–f). CEOF mode-1 and 2 contributed the dominant features during the post-monsoon season (i.e. October, Fig. 7 k,q). However, the dominant transport patterns are not seen all along the west coast of India, as illustrated by the cLCS. During October, the EICC is strong and flows equatorward, which is clearly captured in cLCS along India's west coast (Fig. 7e). Thus, the cLCS associated with the monsoon-induced coastal current (EICC) and the eddies over the western BoB clearly shows the dominant transport pattern. But only during July and August, the cLCS over eastern BoB and southeastern BoB not as significant as EOFs. Overall, this section described the dominant transport patterns that can be used to predict pollutant transport by augmenting the cLCS and CEOFs.

#### Validation of derived cLCSs for the oil spill trajectory prediction

The recently discovered Lagrangian coherent structures (LCSs) are the hidden skeletons of complex mixing patterns in unsteady flows to make short-term predictions. One of the major oil spill events in history that occurred in the Gulf of Mexico (Deep Water Horizon oil spill) was accurately predicted through the LCS core analysis^21,78^. In this study, an attempt has been made to depict the capability of cLCSs in predicting the oil spill trajectory in the BoB. The Liquefied Petroleum Gas tanker BW Maple and the chemical tanker MT Dawn Kanchipuram collided about two nautical miles off Kamarajar Port, Ennore, on January 28, 2017, resulting in the spillage of 196.4 metric tonnes of Heavy Furnace Oil^9^.

**Figure 8:**
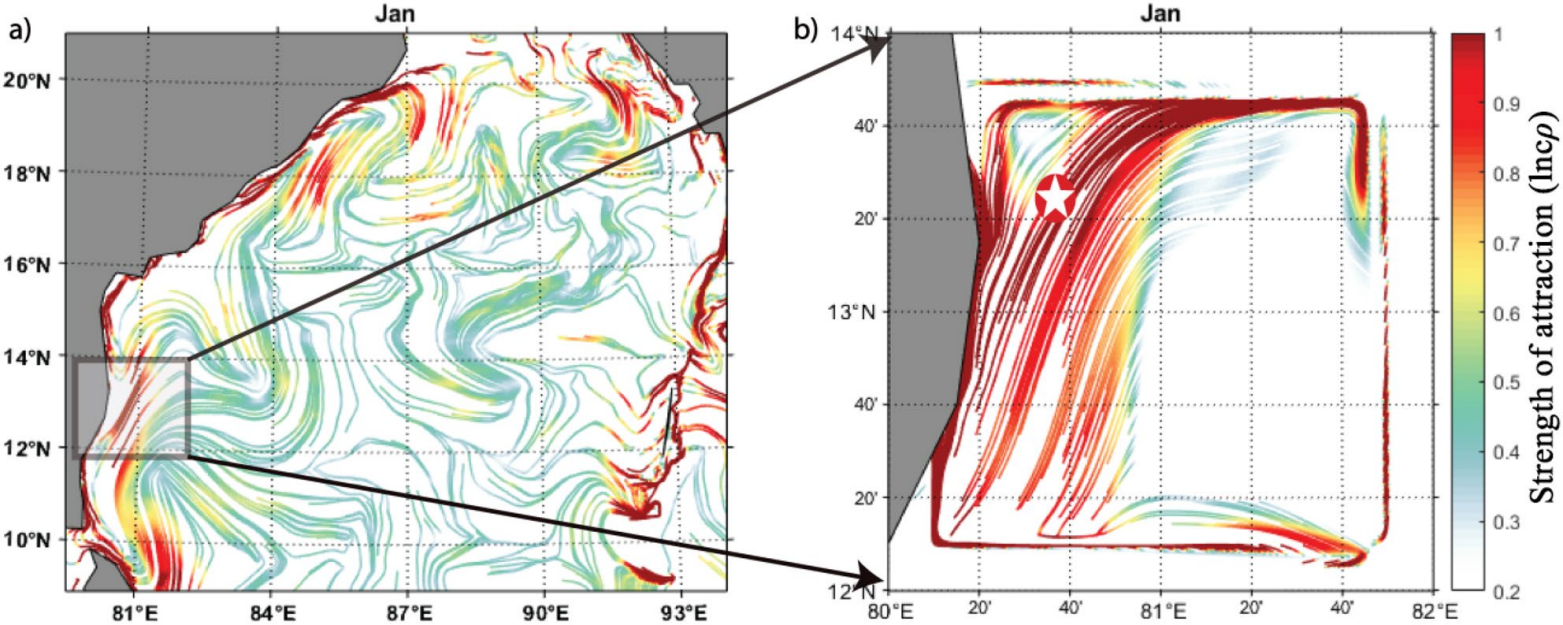
(**a**) The basin-wide cLCS pattern over the Bay of Bengal during January and (**b**) Subset indicate the spill incident area, the zoomed view of square box in Fig. (**a**). (The colour bar in the figure represents the variation of climatological attraction strengths in lncρ).

**Figure 9:**
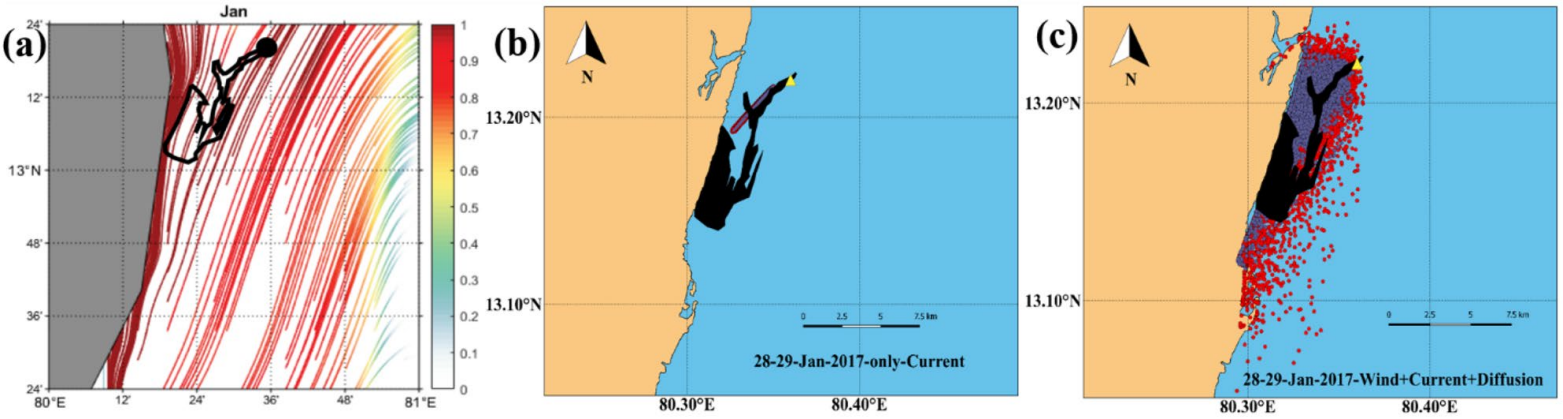
(**a**). Climatological LCSs (The colour bar in the figure represents the variation of climatological attraction strengths in lncρ) for January overlaid with Sentinel-1 shape file image (black colour dot indicates spill location and a black shape file for spill spreading). (**b**) GNOME oil spill model trajectory (purple line) simulated with only currents overlay with the SAR observed oil slick shapefile (solid black). (**c**). GNOME oil spill model trajectory (red dots) simulated with winds, currents, and diffusion coefficient overlay with the SAR observed oil slick shapefile (solid black). The purple dots show the best guess.

The GNOME-based oil spill trajectory prediction shows the southward drift of the spill, which has perfectly matched the satellite sentinel-1 imagery and the in situ observations made by the Indian Coast Guard^9^. Therefore, the computed cLCSs over the BoB are compared with this GNOME simulated oil spill trajectory. Figure 8a depicts the basin-wide cLCS pattern during January. As discussed above, robust and attractive cLCSs are seen along India's east coast. The zoomed view of cLCSs is depicted in Fig. 8b. The strong, attractive streamlines flow along the Tamil Nadu State coastal region with a slight curve offshore in its northern part. The spill origin exactly falls on these strong, attractive streamlines. The predominant Lagrangian transport streamlines flowing southwestward suggest that the spill would be moved along their path and could reach the coast (Fig. 8b). The oil spill incident occurred on January 28, 2017, but the sentinel-1 satellite passed through this spill region one day later, i. e the available sentinel image was on January 29, 2017 (ID: 29–01-2017-6D04). The image was obtained from the sentinel scientific data hub and processed through the sentinel toolbox (SNAP) using the image processing method^63^. Figure 9 is the final processed output shape file image overlaid on cLCSs, wherein the black colour dot indicates the spill origin.

The satellite sentinel-1 captured the spill drift pattern on January 29 2017 (Fig. 9a). It shows that the spill has drifted southwestward from its origin and reached the shore, as illustrated in Fig. 9a. The affected coast length is ~ 7 km, and the area of spill spreading is ~ 13.5 km^2^. Figure 9b depicts the GNOME simulated (currents alone) trajectory (purple) overlay with the satellite-captured oil slick shape file. The GNOME trajectory indicates that the spill is drifting southeastward and likely to reach the coast. Therefore, the LCS, and the GNOME trajectory (with currents) indicate that the predominant transport of the oil spill is southeastward and reaches the coast. These LCS and GNOME trajectories have depicted the likely destination of the oil spill, although they have not followed the exact trajectory as seen in satellite imagery. Nevertheless, Fig. 9c depicts the GNOME simulated (with winds, currents, and diffusion) overlay with the satellite-captured oil spill drift. The trajectory matches relatively better with the satellite-captured oil spill, indicating that including wind can improve the oil spill trajectory. Nevertheless, the cLCS can be helpful to immediately understand the destination of the spill, although its trajectory does not strictly follow the actual spill drift. Recently, Gouveia et al.^12^ have shown the cLCS capability in extracting the predominant transport patterns that help explain the observed drift of Chevron's oil spill (November 2011) and the recent oil spills observed at Brazilian beaches (from August 2019 to February 2020). Our study shows that it is acceptable that the extracted persistent cLCSs perform well and can be utilized to predict the trajectories of any floating pollutants such as oil spills, plastic debris etc., and the inclusion of wind effect will further improve its trajectory.

#### Application of derived cLCS maps on the transport of hypothetical oil spills near Krishna-Godavari basin (KG basin)

This section describes the drift or transport of hypothetical oil spills that occur over the Krishna Godavari (KG) basin with perceived monthly climatological LCS maps. KG Basin is one of the proven petroliferous basins situated on the passive margin^79^ on the east coast of India. Several oil and gas fields are established onshore and offshore in this basin. Besides the oil explorations, major ship routes are connected from coastal India to different countries such as Sri Lanka, Bangladesh, Singapore, Myanmar, Thailand, and Sumatra.

**Figure 10:**
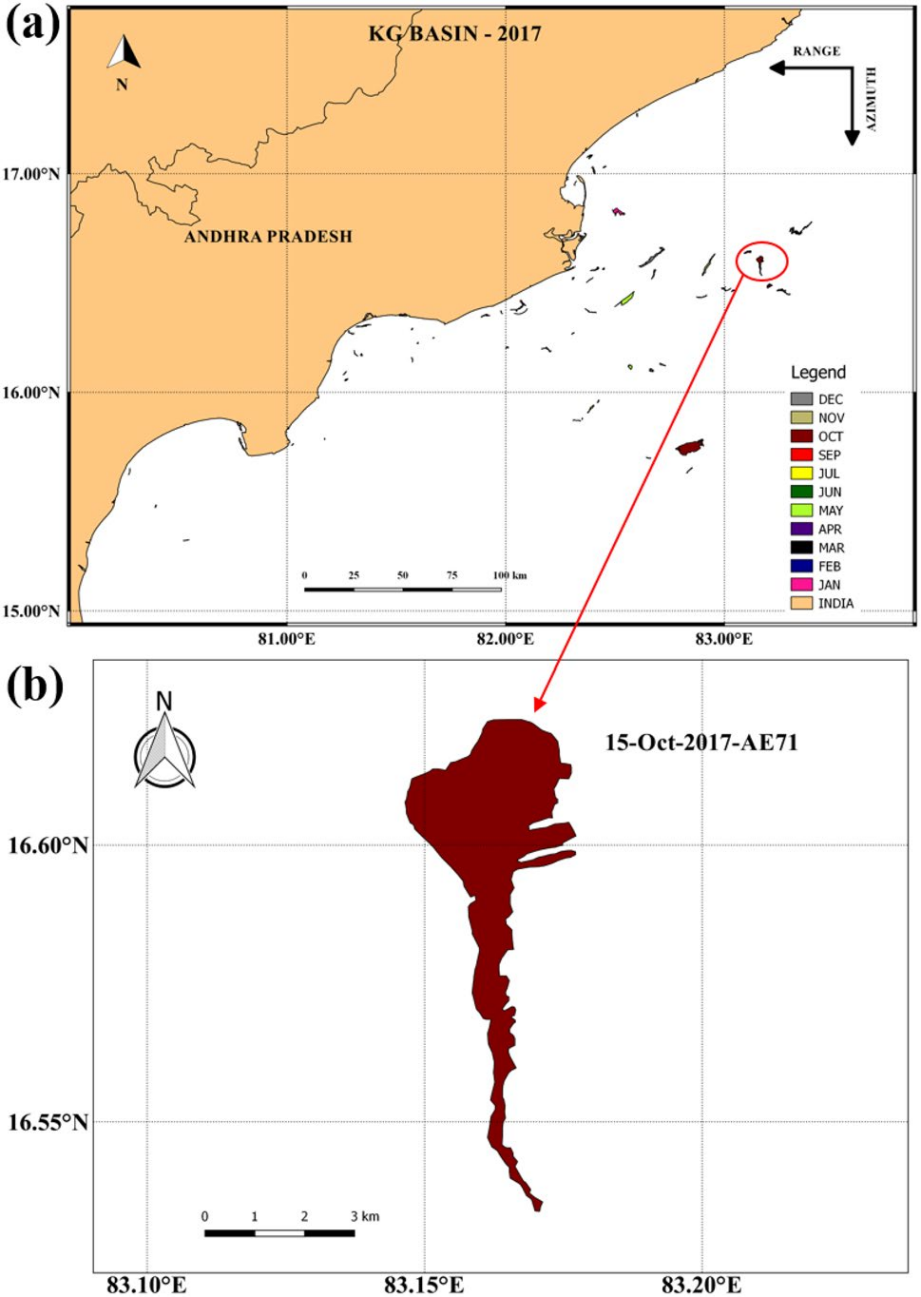
(**a**) Distribution of oil spills along the East Coast of India (KG-Basin) in 2017, (**b**) Oil spill shapefile image on October 15, 2017 (ID No. AE71).

Since the KG basin is one of the active oil exploration sites/shipping activity along the east coast of India, the occurrence of oil spills may be feasible at any time. Therefore, sentinel-1 imagery is used for one year (2017) to verify the occurrence of oil spills in this region. The results shown in Fig. 8a illustrate that there are few oil spills caused primarily by ship traffic. However, there is one spill that occurred on October 15 (ID No. AE71) which does not seem to be derived from the ships but resembles a natural leakage (broader origin and a narrow tale) (Fig. 10a,b). Thus, based on this image, although it is not confirmed that it is natural seepage, this location has been chosen as a hypothetical oil spill origin and studied the likely movement of oil spills with respect to each calendar month based on the LCS maps to primarily depict the vulnerable months in which the spill can reach to the shoreline. The black arrows overlaid with cLCS show the mean direction of the cLCS pattern.

**Figure 11:**
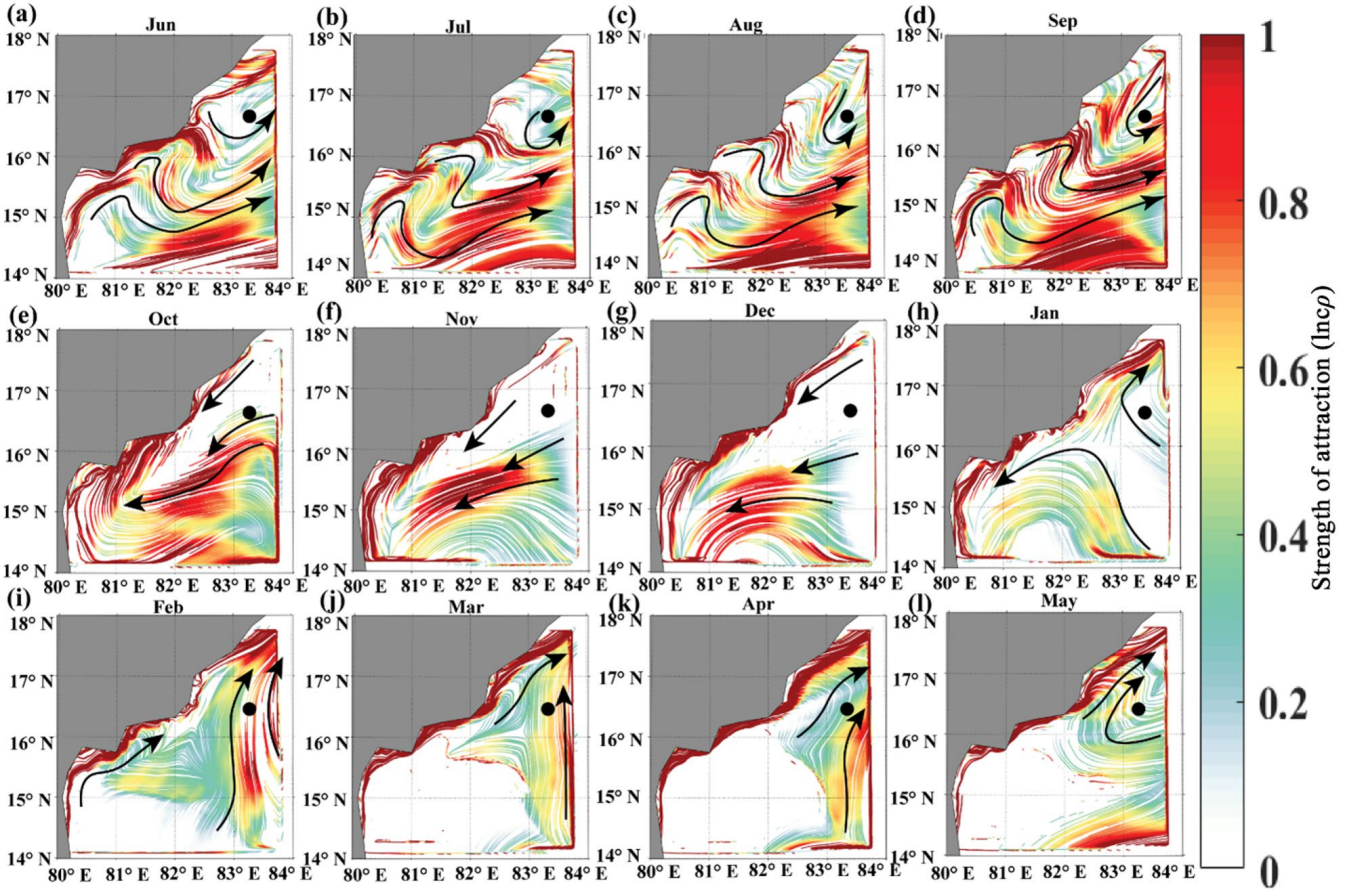
Observed monthly climatological (1994–2017) cLCS (The colour bar in the figure represents the variation of climatological attraction strengths in lncρ) patterns near KG-Basin overlaid by the mean current direction (black arrows demark flow pattern), and the dark circle show the hypothetical oil spill origin. Panels a, b, c and d represent the southwest monsoon, e, f, g, h, the northeast monsoon, and i, j, k, and l represent pre-monsoon seasons.

Figure 11a,b,c, and d show that a highly intensified strong, attractive cLCS pattern is observed near KG-basin during June, July, August, and September (Southwest monsoon season) months. During the southwest monsoon season, the monsoon-induced EICC flows towards the poles but slightly northeastward. Thus, any oil spill near the KG basin during the southwest monsoon season will drift away from the coast towards the central BoB. Therefore, during this season, there is no threat of oil pollution to the coastal region of the KG basin.

As described in “cLCS during the Indian winter monsoon period” the circulation pattern over BoB is in an anticlockwise direction, and the EICC flows equatorward during the northeast monsoon (October to February)^69^. The Fig. 11e,f, and g illustrate the strong south-southeastward cLCS pattern during October, November, and December. These cLCS maps overlaid with the mean current direction show the southward transport pattern during this season (OND). During January, the EICC changed its direction off the Andhra Coast; one branch is going to the south, and the other is going north. Hence, the corresponding cLCS are also vital along the coast (Fig. 11h). The comparison between the simulated oil spill and cLCSs maps shows reasonable agreement during this period (Fig. 9). Therefore, the strongly attracted southward cLCSs indicate a high chance of oil spill reaching the shore if any occurs in the KG basin during this season. The coastal habitats near the coastline will be under threat in this season.

The cLCS maps overlaid with the mean current direction during February, March, April, and May (Fig. 11i, j, k, and l) indicate that the coastal region north of the KG basin (north Andhra Pradesh or the Odisha state coast) may likely be affected by the oil spills if any spills occur in the vicinity of KG basin. Overall, the cLCS monthly maps illustrate that from June to September, only the spills will not reach the shore, whereas, for the rest of the year, the spill may likely reach the shore. We recommend that these cLCS maps are well-suitable for oil spill monitoring and prediction for instant rescue operations along the coastal regions.

## Summary and conclusion

Persistent climatological Lagrangian coherent structures (cLCS) (surface transport patterns) were extracted from the computed long-term (24 years) surface velocity fields in the Bay of Bengal for the first time. The LCSs were compared with the satellite drifter data and found to be reasonably matching other than the sub-mesoscale activity region. The potential monthly cLCS maps in response to the SW monsoon, NE monsoon, and pre and post-monsoon periods were discussed. Further, this study also depicted and demonstrated the cLCS trends associated with the strong western boundary current (EICC) and the mesoscale processes (eddies) in the BoB. The results show one seasonal accumulation zone off Visakhapatnam, wherein the cLCS are strongly associated with the seasonal anticyclonic eddy. Thus, any pollutants from the strong coastal currents may get trapped in this zone. The robust, attractive cLCS are seen along the coastal boundary of the BoB throughout the year compared to the open ocean. Overall, the strong, attractive coastal cLCS are yielded in response to the monsoon-induced coastal currents and their associated mesoscale processes, such as eddies. An attempt has been made to unravel the impact of winds on the cLCS. The results indicated that the wind influence is moderately seen only during November- December. Interestingly, the computed cLCSs maps also depicted the transport pattern of the northern BoB freshwater plume southward along the east coast of India, indicating that these cLCS maps can also give meaningful information on freshwater plume transport.

In the current study, we also used the CEOF analysis to confirm the dominant features depicted by the cLCS and to reveal any features that the cLCS do not capture. The first and second modes of CEOF variability mainly contributed to the dominant features of surface transport, as revealed by the cLCS. The results show that the dominant transport pattern induced by the monsoon currents and eddies is more significant in cLCS than in EOFs. However, during July and August, a few transport patterns are more significant in EOFs than in cLCS. To our knowledge, this is the first approach that has been used in the BoB to reveal surface transport patterns by augmenting the cLCS and EOFs. The application of these cLCSs maps in pollutant trajectory prediction is also verified with the actual oil spill incident of Ennore Port, Chennai. The results match each other well and depict the oil spill's trajectory. This is an excellent method to ensure pollutant dispersion and movement in the region of the spill incident area. These extracted persistent cLCSs are exactly apt for the dispersion of the oil spill movement. Considering the hypothetical oil spills at one of the oil exploration sites, the KG basin, we discussed the transport of the oil spills based on the computed cLCS maps. The results indicated that other than the southwest monsoon season, the coastal region of Andhra Pradesh coast is under threat due to oil spills. We confirm that cLCSs extract the kinematics from the multiyear velocity time series, giving understandable synthesized information. These climatological maps provide valuable information complementing oil spill forecasts (Barker 2011). The great advantage is that these maps help forecast the trajectory of oil spills or any surface floating pollutants caused by any accidents/incidents in the Bay of Bengal in the future.

We have utilized the same HYCOM currents used for LCS to simulate the GNOME model. Randomly, the year 2015 was chosen to track the tracer (oil particles) dispersion in the Bay of Bengal at three locations. The particles released at L1 and L3 have closely followed the cLCS trajectories. However, a slight deviation of GNOME particles is seen at L2 (Figure. S3). This could be due to the windage effect considered in GNOME but absent in cLCS. Thus, once these climatological Lagrangian Coherent Structures are available, they act as a primary proxy to identify the spill trajectory, mitigate the risk, and are helpful in planning the immediate response. However, there are a couple of limitations to be noted. The surface currents used for LCS should include the wind effect. Also, the direction of the LCS fields will not be known to readers who do not have background knowledge of the seasonal reversal of the current system in the Bay of Bengal. Thus, basic knowledge of the background Eulerian flow field is necessary to understand the LCS direction. However, it is to be noted that as indicated by the Duran et al. 2018, these LCS maps are only the added advantage for the quick prediction of pollutant trajectories and not the substitute for the instantaneous velocity predictions.

### Supplementary Information

Supplementary Information.

## Data Availability

The zonal and meridional wind velocity at 10 m height were obtained from the ECMWF ERA5 (https://cds.climate.copernicus.eu/cdsapp#!/dataset/reanalysis-era5-single-levels?tab=form), and the zonal and meridional current velocities are obtained from the site https:/hycom.org/. The SMAP SSS data were obtained from https://podaac.jpl.nasa.gov/dataset/SMAP_JPL_L3_SSS_CAP_8DAY-RUNNINGMEAN_V43). The sentinel-1 satellite data were obtained from the Copernicus Open Access Hub (https://www.copernicus.eu/en). The computation of cLCS was performed using the Matlab codes available at https://bitbucket.org/rodu/clcss. The surface drifter data are obtained from https://www.aoml.noaa.gov/phod/gdp/hourly_data.php.

## References

[1] Mera D, Cotos JM, Varela-Pet J, Garcia-Pineda O (2012). Adaptive thresholding algorithm based on SAR images and wind data to segment oil spills along the northwest coast of the Iberian Peninsula. Mar. Pollut. Bull..

[2] Ivshina IB (2015). Oil spill problems and sustainable response strategies through new technologies. Environ. Sci. Process Impacts..

[3] Rajendran S (2021). Detection of Wakashio oil spill off Mauritius using Sentinel-1 and 2 data: Capability of sensors, image transformation methods, and mapping. Environ. Pollut..

[4] Gurumoorthi K, Suneel V, Trinadha Rao V, Thomas AP, Alex MJ (2021). Fate of MV Wakashio oil spill off Mauritius coast through modelling and remote sensing observations. Mar. Pollut. Bull..

[5] Trinadha Rao V, Suneel V, Gurumoorthi MJ, Thomas AP (2022). Assessment of MV Wakashio oil spill off Mauritius, Indian Ocean through satellite imagery: A case study. J. Earth Syst. Sci..

[6] Liu F, Liu J, Chen Q, Wang B, Cao Z (2013). Pollution characteristics and ecological risk of polycyclic aromatic hydrocarbons (PAHs) in surface sediments of the southern part of the Haihe River system in China. Chin. Sci. Bull..

[7] Desa E (2005). Dissolved oxygen––A target indicator in determining use of the Gulf of Kachchh waters. Mar. Pollut. Bull..

[8] Vethamony P (2007). Trajectory of an oil spill off Goa, eastern Arabian Sea: Field observations and simulations. Environ. Pollut..

[9] Prasad SJ, Balakrishnan Nair TM, Rahaman H, Shenoi SSC, Vijayalakshmi T (2018). An assessment on oil spill trajectory prediction: Case study on oil spill off Ennore Port. J. Earth Syst. Sci..

[10] Zodiatis G, Carpenter A (2018). Numerical modeling of oil pollution in the Eastern Mediterranean Sea. Oil Pollution in the Mediterranean Sea: Part I: The International Context.

[11] García-Garrido VJ, Ramos A, Mancho AM, Coca J, Wiggins S (2016). A dynamical systems perspective for a real-time response to a marine oil spill. Mar. Pollut. Bull..

[12] Gouveia MB (2021). Persistent meanders and eddies lead to quasi-steady Lagrangian transport patterns in a weak western boundary current. Sci. Rep..

[13] Provenzale A (1999). Transport by coherent barotropic vortices. Annu. Rev. Fluid Mech..

[14] Boffetta G, Lacorata G, Redaelli G, Vulpiani A (2001). Detecting barriers to transport: a review of different techniques. Phys. D: Nonlinear Phenom.

[15] Peacock T, Dabiri J (2010). Introduction to focus issue: Lagrangian coherent structures. Chaos: An Interdisciplinary. J. Nonlinear Sci..

[16] Broer HW, Osinga HM, Vegter G (1997). Algorithms for computing normally hyperbolic invariant manifolds. Z. Angew. Math. Phys..

[17] Haller G (2001). Distinguished material surfaces and coherent structures in three-dimensional fluid flows. Phys. D Nonlinear Phenom.

[18] Shadden SC, Lekien F, Marsden JE (2005). Definition and properties of Lagrangian coherent structures from finite-time Lyapunov exponents in two-dimensional aperiodic flows. Phys. D Nonlinear Phenom.

[19] Gough MK (2016). Lagrangian Coherent Structures in a coastal upwelling environment. Conti. Shelf Res..

[20] Olascoaga MJ, Haller G (2012). Forecasting sudden changes in environmental pollution patterns. Proc. Natl. Acad. Sci. USA.

[21] Olascoaga MJ (2013). Drifter motion in the Gulf of Mexico constrained by altimetric Lagrangian coherent structures. Geophys. Res. Lett..

[22] Beron-Vera FJ, LaCasce JH (2016). Statistics of simulated and observed pair separations in the Gulf of Mexico. J. Phys. Oceanogr..

[23] Haza AC, Ozgokmen T, Hogan P (2016). Imapct of submesoscales on surface material distribution in a gulf of Mexico mesoscale eddy. Ocean Modell..

[24] García-Sánchez G, Mancho AM, Ramos AG, Coca J, Wiggins S (2022). Structured pathways in the turbulence organizing recent oil spill events in the Eastern Mediterranean. Sci. Rep..

[25] Duran R, Beron-Vera FJ, Olascoaga MJ (2018). Extracting quasi-steady Lagrangian transport patterns from the ocean circulation: An application to the Gulf of Mexico. Sci. Rep..

[26] Houseago-Stokes RE, Challenor PG (2004). Using PPCA to estimate EOFs in the presence of missing values. J. Atmos. Ocean Technol..

[27] Beckers JM, Rixen M (2003). EOF calculations and data filling from incomplete oceanographic datasets. J. Atmos. Ocean Technol..

[28] Henn B, Raleigh MS, Fisher A, Lundquist JD (2013). A comparison of methods for filling gaps in hourly near-surface air temperature data. J. Hydrometeorol..

[29] Taylor MH, Losch M, Wenzel M, Schro¨ter J (2013). On the sensitivity of field reconstruction and prediction using empirical orthogonal functions derived from Gappy data. J. Clim..

[30] Hardy DM (1977). Empirical eigenvector analysis of vector observations. Geophys. Res. Lett..

[31] Legler DM (1983). Empirical orthogonal function analysis of wind vectors over the tropical Pacific region. Bull. Amer. Meteor. Soc.

[32] Kundu PK, Allen JS (1976). Some three-dimensional characteristics of low-frequency current fluctuations near the Oregon coast. J. Phys. Oceanogr..

[33] Klinck JM (1985). EOF analysis of central drake passage currents from DRAKE 79. J. Phys. Oceanogr..

[34] Prandle D, Matthews J (1990). The dynamics of nearshore surface currents generated by tides, wind and horizontal density gradients. Cont. Shelf Res..

[35] Ng B (1993). The prediction of nearshore wind-induced surface currents from wind velocities measured at nearby land stations. J. Phys. Oceanogr..

[36] Kutzbach JE (1967). Empirical eigenvectors of sea-level pressure, surface temperature and precipitation complexes over North America. J Appl Meteorol Climatol..

[37] Shankar D, Vinayachandran PN, Unnikrishnan AS (2002). The monsoon currents in the north Indian Ocean. Prog. Oceanogr..

[38] Shetye SR (1993). The western boundary current of the seasonal subtropical gyre in the Bay of Bengal. J. Geophys. Res. Oceans..

[39] Shenoi SSC, Antony MK (1991). Current measurements over the western continental shelf of India. Cont. Shelf Res..

[40] Shankar D, McCreary JP, Han W, Shetye SR (1996). Dynamics of the East India Coastal Current: 1. Analytic solutions forced by interior Ekman pumping and local alongshore winds. J. Geophys. Res. Oceans.

[41] Shetye SR, Gouveia AD (1998). Coastal circulation in the north Indian Ocean: Coastal segment (14, SW).

[42] Schott F, McCreary JP (2001). The monsoon circulation of the Indian Ocean. Prog. Oceanogr..

[43] Durand F, Shankar D, Birol F, Shenoi SSC (2009). Spatiotemporal structure of the East India Coastal Current from satellite altimetry. J. Geophys. Res. Ocean..

[44] Dandapat S, Chakraborty A, Kuttippurath J (2018). Interannual variability and characteristics of the east india coastal current associated with indian ocean dipole events using a high resolution regional ocean model. Ocean Dyn..

[45] Das BK, Anandh TS, Kuttippurath J, Chakraborty A (2019). Characteristics of the discontinuity of western boundary current in the Bay of Bengal. J. Geophys. Res. Ocean.

[46] Haller G (2015). Lagrangian coherent structures. Annu. Rev. Fluid Mech.

[47] Gough MK, Beron-Vera FJ, Olascoaga JS, Jouanno J, Duran R (2019). Persistent Lagrangian transport patterns in the northwestern Gulf of Mexico. J. Phys. Oceanogr..

[48] Kolukula SS, Baduru B, Murty PLN, Kumar JP, Rao EPR, Shenoi SSC (2020). Gaps filling in HF radar sea surface current data using complex empirical orthogonal functions. Pure Appl. Geophys..

[49] Kaihatu JM, Handler RA, Marmorino GO, Shay LK (1998). Empirical orthogonal function analysis of ocean surface currents using complex and real-vector methods. J Atmos Ocean Technol..

[50] Hardy DM, Walton JJ (1978). Principal components analysis of vector wind measurements. J. Appl. Meteorol. Clim..

[51] Brink KH, Muench RD (1986). Circulation in the point conception-Santa Barbara channel region. J. Geophys. Res. Oceans..

[52] Maslo A, Correia A, de Souza JM, Andrade-Canto F, Rodríguez Outerelo J (2020). Connectivity of deep waters in the Gulf of Mexico. J. Mar. Syst..

[53] Bleck R (2002). An oceanic general circulation model framed in hybrid isopycnic-Cartesian coordinates. Ocean Modell..

[54] Yao F, Johns WE (2010). A HYCOM modeling study of the Persian Gulf: 2 formation and export of persian Gulf Water. J. Geophys. Res. Ocean.

[55] Mezić I, Loire S, Fonoberov VA, Hogan P (2010). A new mixing diagnostic and Gulf oil spill movement. Science.

[56] Mariano MJ, Villano R, Fleming E (2011). Technical efficiency of rice farms in different agroclimatic zones in the Philippines: An application of a stochastic metafrontier model. Asian Econ. Pap.

[57] Periáñez Rodríguez R, Pascual Granged AJ (2008). Modelling surface radioactive, chemical and oil spills in the Strait of Gibraltar. Comput. Geosci.

[58] Pradhan B, Das M, Pradhan C (2022). Trajectory modelling for hypothetical oil spill in Odisha offshore India. J. Earth Syst. Sci..

[59] Makatounis PEZ, Stamou AI, Ventikos NP (2003). Modeling the Agia Zoni II tanker oil spill in Saronic Gulf Greece. Mar. Pollut. Bull..

[60] Wallcraft, A., Carroll, S. N., Kelly, K. A., & Rushing, K. V. Hybrid Coordinate Ocean Model (HYCOM) Version 2.1 User's Guide. Nav. Res. Lab (2003).

[61] Elipot S, Sykulski A, Lumpkin R, Centurioni L, Pazos M (2022). Hourly location, current velocity, and temperature collected from global drifter program drifters world-wide. Accession.

[62] Duran R, Beron-Vera FJ, Olascoaga MJ (2019). CIAM climatological isolation and attraction model-climatological lagrangian coherent structures. Albany Natl. Energy Technol. Lab.-Energy Data Exch. NETL..

[63] Suneel V (2019). Oil pollution in the Eastern Arabian Sea from invisible sources: A multi-technique approach. Mar. Poll. Bull..

[64] Boufadel MC, Abdollahi-Nasab A, Geng X, Galt J, Torlapati J (2014). Simulation of the landfall of the Deepwater Horizon oil on the shorelines of the Gulf of Mexico. Environ. Sci. Technol..

[65] Zelenke, B., O'Connor, C., Barker, C. H., Beegle-Krause, C. J., & Eclipse, L. General NOAA operational modeling environment (GNOME) technical documentation. (2012). NOAA technical memorandum NOS-OR&R 40

[66] Hersbach H (2018). ERA5 hourly data on single levels from 1979 to present. Copernicus Climate Change Service Climate Data Store.

[67] Hersbach H (2020). The ERA5 global reanalysis. Q. J. R. Meteorol. Soc..

[68] Shetye SR (1996). Hydrography and circulation in the western Bay of Bengal during the Northeast Monsoon. J. Geophys. Res..

[69] Mukherjee A (2014). Observed seasonal and intraseasonal variability of the East India coastal current on the continental slope. J. Earth Syst. Sci..

[70] Dandapat S, Chakraborty A (2016). Mesoscale eddies in the Western Bay of Bengal as observed from satellite altimetry in 1993–2014: Statistical characteristics variability and three-dimensional properties. IEEE J. Sel. Top. Appl. Earth Obs. Remote Sens..

[71] Shenoi SSC, Shankar D, Shetye SR (2002). Differences in heat budgets of the near-surface Arabian Sea and Bay of Bengal: Implications for the summer monsoon. J. Geophys. Res. Oceans.

[72] Suneel V (2020). Impact of remote equatorial winds and local mesoscale eddies on the existence of River in the Sea along the East coast of India inferred from satellite SMAP. J. Geophys. Res. Oceans..

[73] Chaitanya AVS (2014). Salinity measurements collected by fishermen reveal a river in the sea flowing along the eastern coast of India. Bull. Am. Meteorol. Soc..

[74] Mathur M, David MJ, Sharma R, Agarwal N (2019). Thermal fronts and attracting lagrangian coherent structures in the north Bay of bengal during december 2015–march 2016. Deep Sea Res. Part II Top Stud. Oceanogr.

[75] Ratheesh S (2020). Response of a high-resolution ocean circulation model to winds from different sources in simulating summer monsoon freshening in the North Bay of Bengal: A case study. Deep Sea Research Part II: Top. Stud. Oceanogr..

[76] Potemra JT, Luther ME, O'Brien JJ (1991). The seasonal circulation of the upper ocean in the Bay of Bengal. J. Geophys. Res. Oceans.

[77] Mukhopadhyay S (2020). Observed variability of the East India coastal current on the continental slope during 2009–2018. J. Earth Syst. Sci..

[78] Haller G, Beron-Vera FJ (2012). Geodesic theory of transport barriers in two-dimensional flows. Phys. D Nonlinear Phenom..

[79] DGH, Krishna Godavari basin 2010. [Online] Available: http:/www.dghindia.org/16.aspx (July 7).

