# Supplementary material for: Extraction of persistent lagrangian coherent structures for the pollutant transport prediction in the Bay of Bengal
Source: Sci Rep. 2024 Apr 16;14:8761. doi: 10.1038/s41598-024-58783-4 (PMC11021457; doi:10.1038/s41598-024-58783-4)
Supplement: Supplementary file 1 — Supplementary Information. [file 41598_2024_58783_MOESM1_ESM.docx]

**Extraction of Persistent Lagrangian Coherent Structures for the Pollutant Transport Prediction in the Bay of Bengal.**

**Trinadha Rao, V.,^1,2,3^ V. Suneel,^*1,2^ Venkata Sai Gulakaram^3^ and Chilukuri Lakshmi Sravani^3^**

**^1^-CSIR-National Institute of Oceanography, Dona Paula - 403004, Goa**

**^2^- Academy of Scientific and Innovative Research (AcSIR), Ghaziabad - 201 002, India**

**^3-^ESSO-Indian National Centre for Ocean Information Services (INCOIS), Ministry of Earth Sciences, Hyderabad 500 090, India.**

A case study has been conducted to understand the discrepancy between the cLCS and the GNOME-based oil spill trajectories. We assumed three oil spills occurred in January 2015 and continued for 15 days (January 1 to January 15). We have used HYCOM currents for the year of January 2015 (15 days) and simulated the oil spill trajectories through the GNOME model. We released 1000 metric tons of oil (tracers) for each location, including the default diffusion (100000 cm2/sec) and windage (3%). The particles released at L1 and L3 have closely followed the cLCS trajectories. However, a slightly different transport of GNOME particles is seen at L2 (Figure. S3). This could be due to the windage effect considered in GNOME but absent in cLCS. This is the one limitation which was already mentioned in the manuscript. Thus, overall, the cLCS can complement the oil spill trajectories to initiate the mitigation measures by the stakeholders quickly.


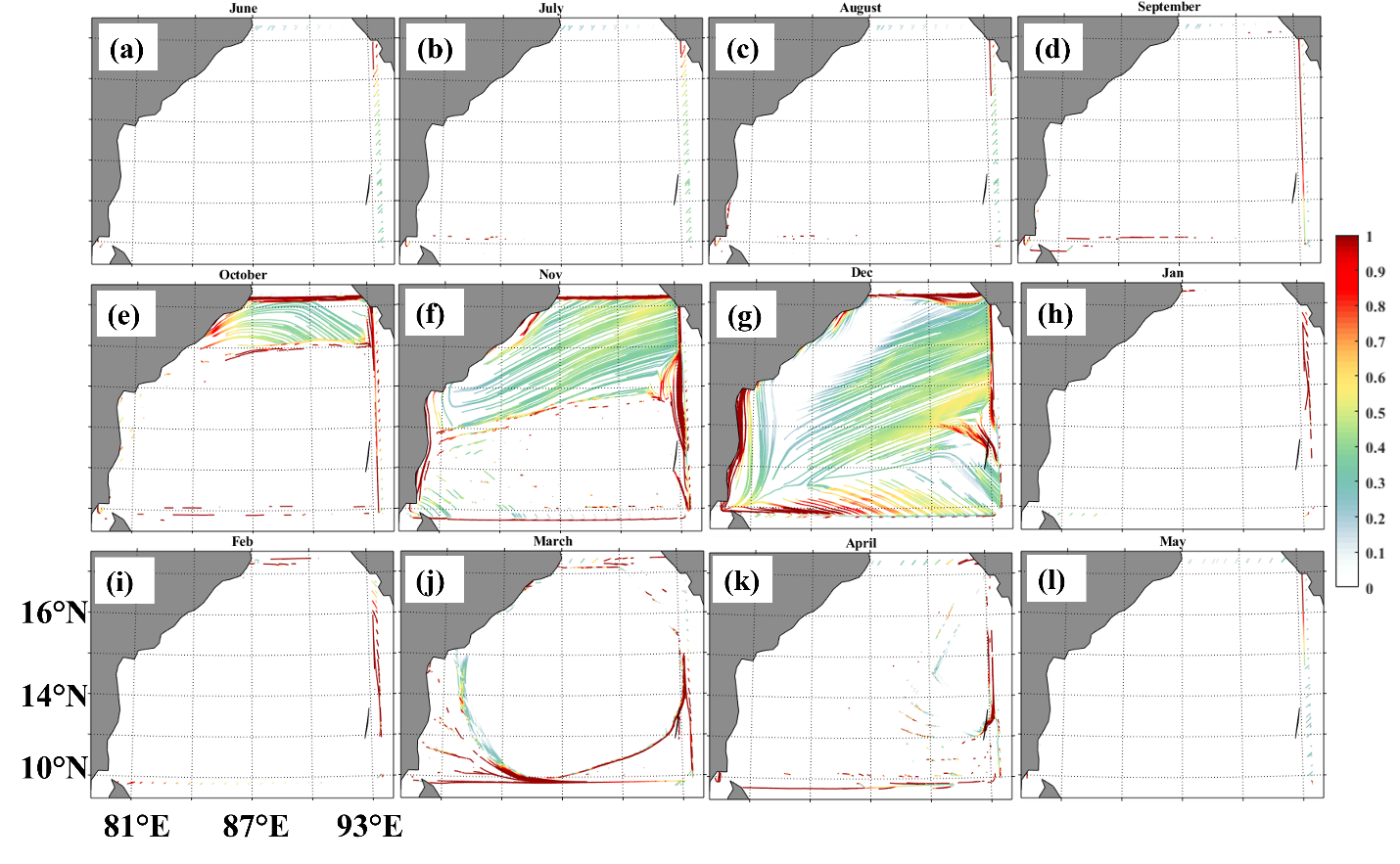


**Figure S1. The monthly climatological Lagrangian Coherent Structures, computed from the surface winds (ECMWF re-analysis winds). June to September (a-d); October to January (e-f); February-May (i-l).**


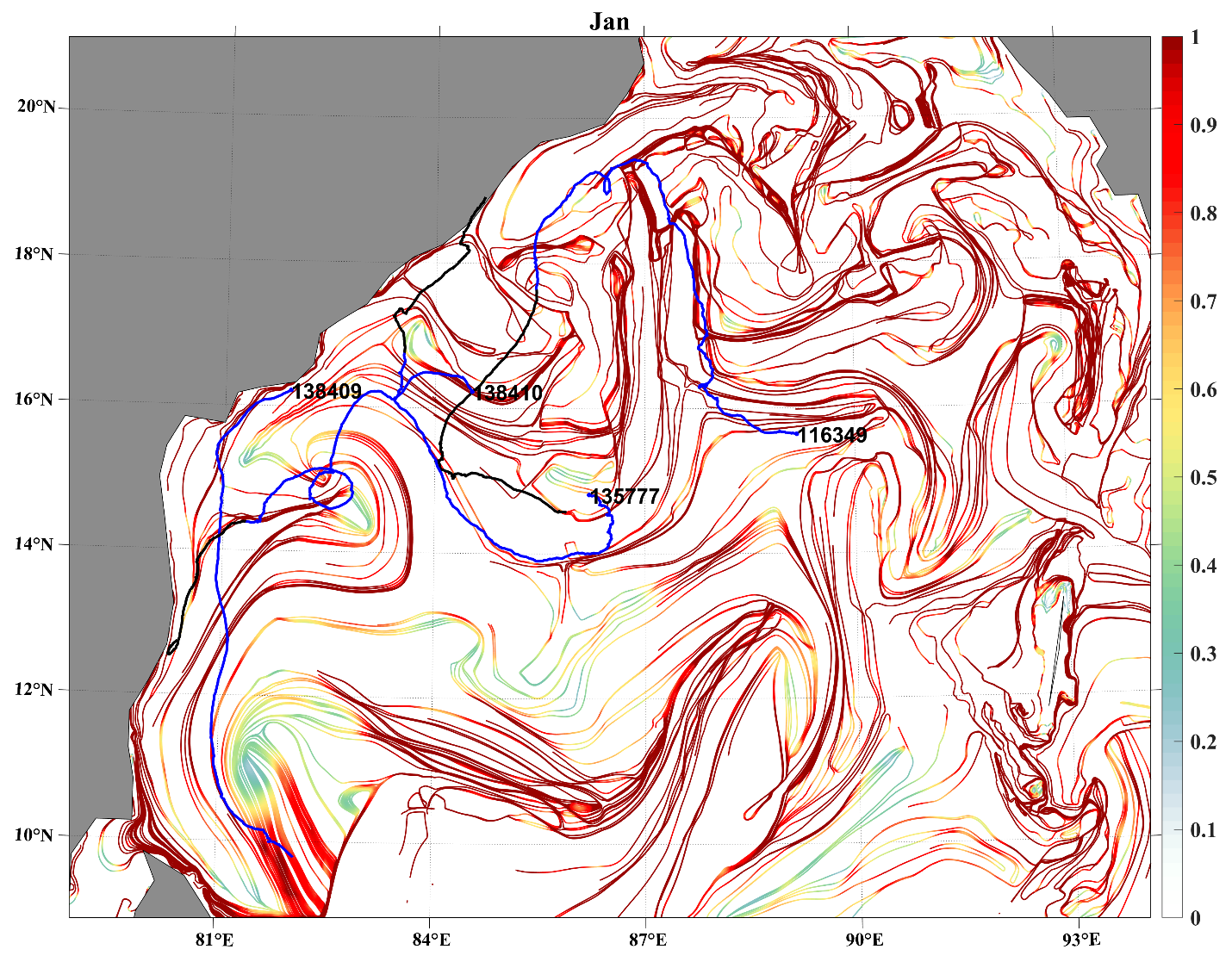


**Figure S2. 2015 January LCS with drifters’ available data, Blue colour represents January month, and black colour represents an extension of January month.**


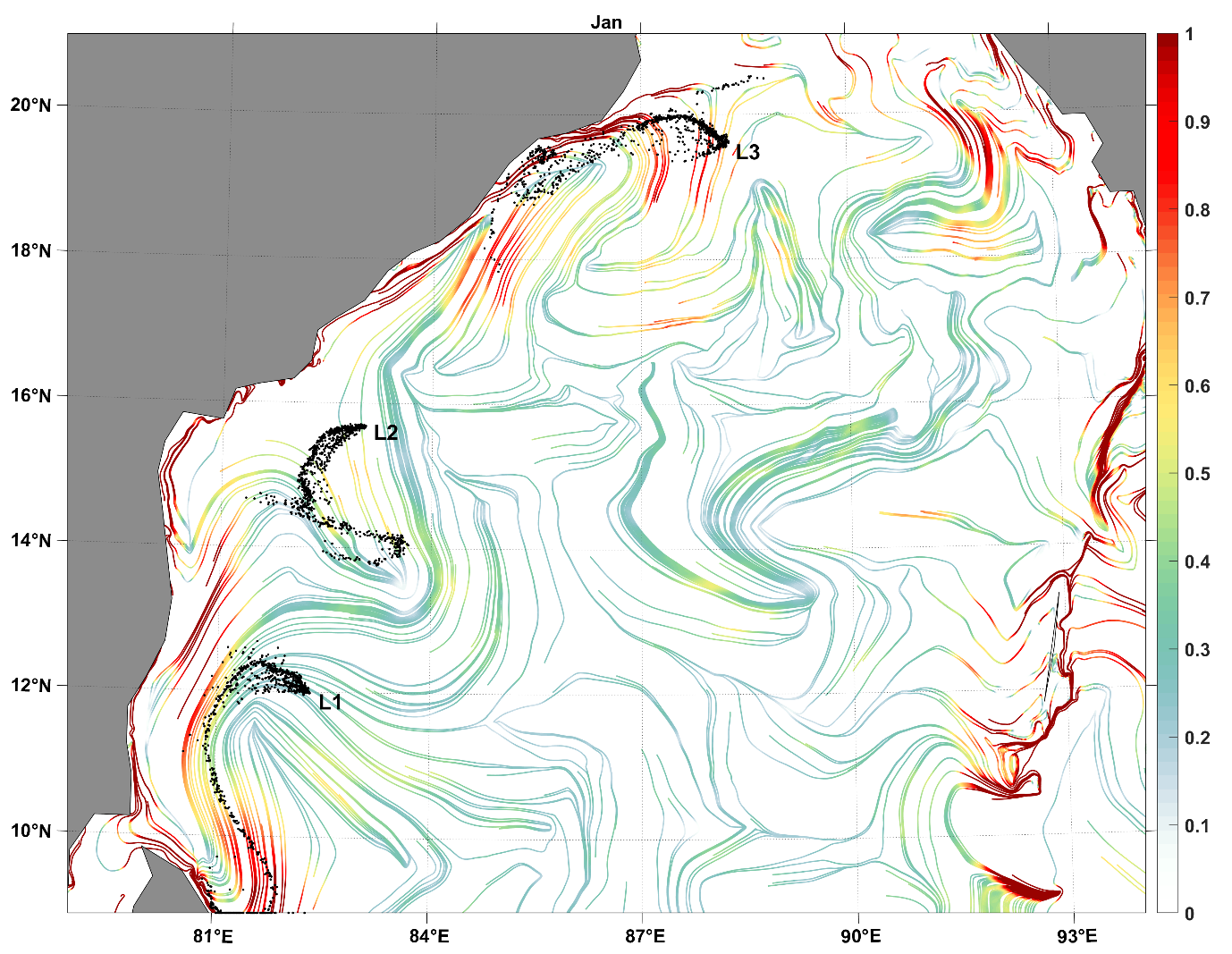


**Figure S3. Climatological Lagrangian Coherent Structures (1994-2017) for January: The black oil splots are three random locations of tracer particle trajectories for January 2015, such as L1, L2, and L3.**
